# Two distinct modes of action of molecular glues in the plant hormone co-receptor COI1-JAZ system

**DOI:** 10.1016/j.isci.2023.108625

**Published:** 2023-12-03

**Authors:** Takuya Kaji, Kotaro Matsumoto, Taichi Okumura, Misuzu Nakayama, Shunji Hoshino, Yousuke Takaoka, Jianxin Wang, Minoru Ueda

**Affiliations:** 1 Department of Chemistry, Graduate School of Science, Tohoku University, Sendai 980-8578, Japan; 2 Department of Molecular and Chemical Life Sciences, Graduate School of Life Science, Tohoku University, Sendai 980-8578, Japan

**Keywords:** Biophysical chemistry, Plant biology, Protein

## Abstract

The plant hormone (3*R*, 7*S*)-jasmonoyl-L-isoleucine ((3*R*, 7*S*)-JA-Ile) is perceived by the COI1-JAZ co-receptor in *Arabidopsis thaliana*, leading to the activation of gene expression for plant defense responses, growth, development, and other processes. Therefore, understanding the interaction between the COI1-JAZ co-receptor and its ligands is essential for the development of COI1-JAZ agonists and antagonists as potent chemical tools for regulating (3*R*, 7*S*)-JA-Ile signaling. This study demonstrated that COI1-JAZ has two independent modes of ligand perception using a differential scanning fluorimetry (DSF) assay. (3*R*, 7*S*)-JA-Ile is perceived through a one-step model in which (3*R*, 7*S*)-JA-Ile causes protein–protein interaction between COI1 and JAZ. In contrast, coronatine (COR), a mimic of (3*R*, 7*S*)-JA-Ile, is perceived through a two-step model in which COR is first perceived by COI1 and then recruits JAZ to form the COI1-COR-JAZ complex. Our results demonstrate two distinct modes of action of molecular glues causing protein–protein interactions.

## Highlights

• The plant hormone (3*R*, 7*S*)-JA-Ile is perceived by the COI1-JAZ co-receptor
• COI1-JAZ has two independent modes of ligand perception
• (3*R*, 7*S*)-JA-Ile is perceived through a one-step perception model
• COR and COR-MO are perceived by two-step perception model

## Graphical abstract

**Figure:**
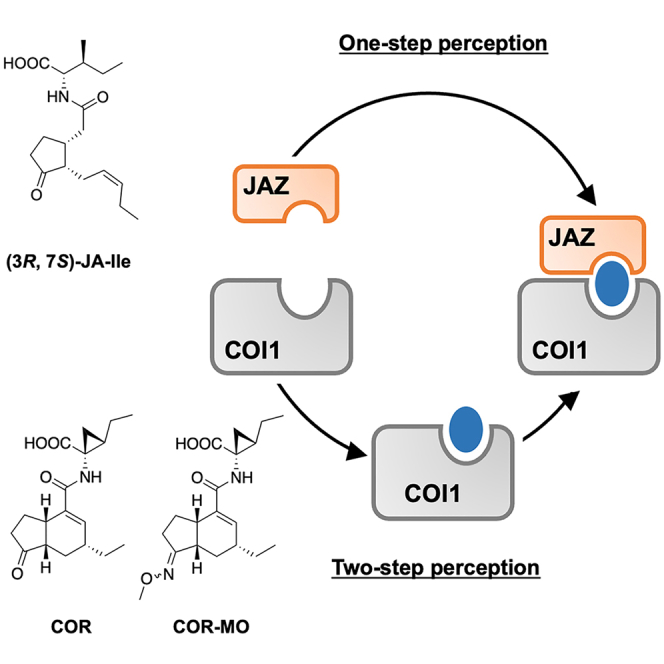

## Introduction

**Figure 1:**
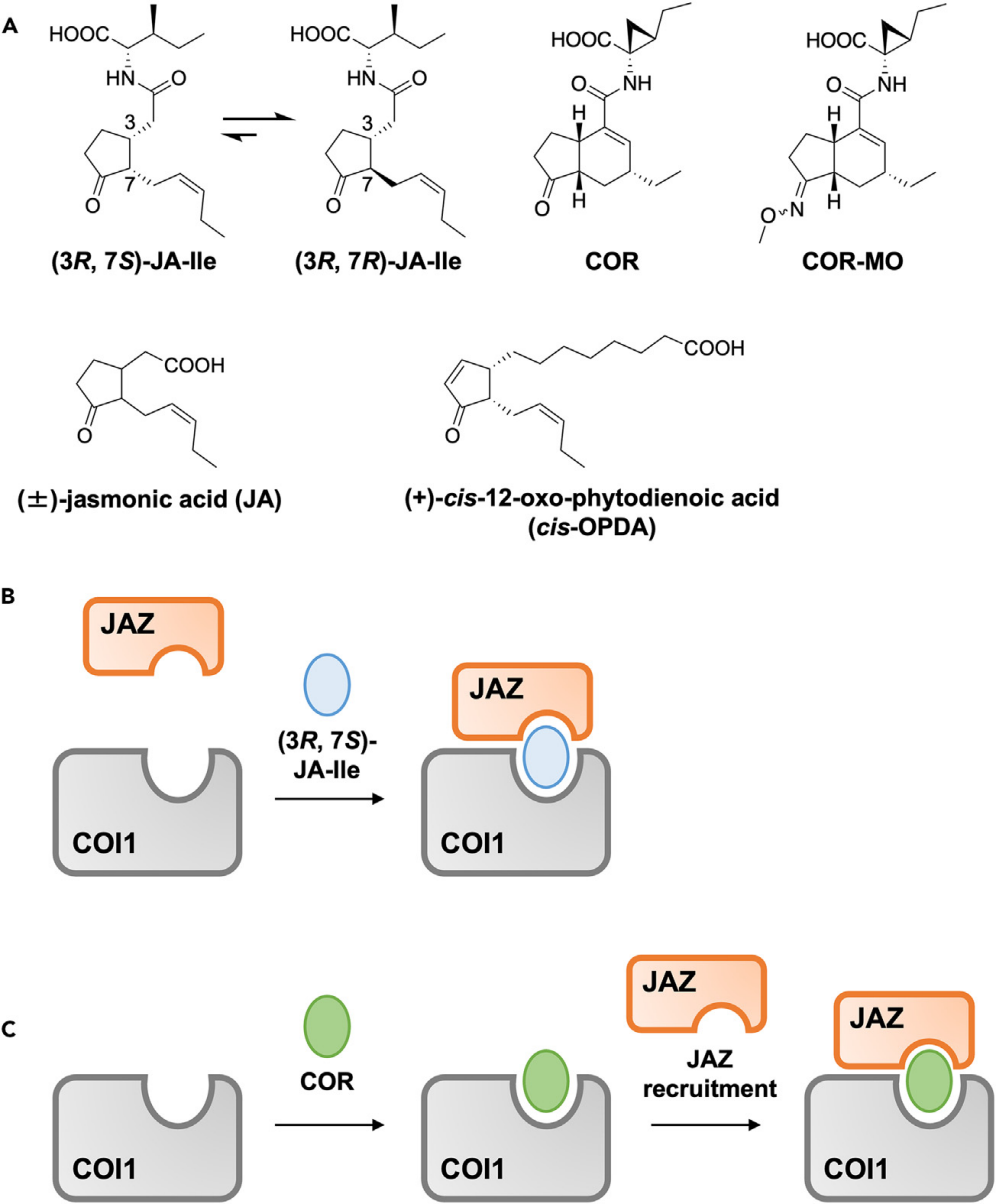
Perception model of JA ligands (A) Chemical structures of JA derivatives. (B) (3*R*, 7*S*)-JA-Ile acts as a molecular glue to induce protein–protein interaction between COI1 and JAZ. (C) COR is first perceived by COI1 and subsequently recruits JAZ to form a COI1-COR-JAZ ternary complex.

(3*R*, 7*S*)-Jasmonoyl-L-isoleucine ((3*R*, 7*S*)-JA-Ile, Figure 1A) is a fatty acid-derived plant hormone that regulates plant defense against pathogens and insects, growth and development, fertility, senescence, secondary metabolite production, and adaptation to environmental stress.^1^^,^^2^^,^^3^^,^^4^^,^^5^ Particularly, JA-Ile is known to activate plant defense response through the regulation of the plant-pathogen and plant-insect interactions at the expense of plant growth.^6^^,^^7^^,^^8^^,^^9^ In *Arabidopsis thaliana*, (3*R*, 7*S*)-JA-Ile causes protein–protein interactions between the F box protein CORONATINE INSENSITIVE 1 (COI1) and jasmonate-ZIM-domain (JAZ) repressor proteins.^10^^,^^11^^,^^12^ The subsequent degradation of the JAZ repressor through the 26S-proteasome mechanism leads to the de-repression of transcription factors, which in turn activates the expression of genes involved in processes such as plant defense responses and growth and development.^1^^,^^2^^,^^4^^,^^5^ The molecular basis of the multiple effects of JA-Ile partially lies in the involvement of the 13 *JAZ* genes encoded in the *Arabidopsis* genome.^1^^,^^13^^,^^14^ Complexation between (3*R*, 7*S*)-JA-Ile and the COI1-JAZ co-receptor is believed to involve (3*R*, 7*S*)-JA-Ile functioning as a molecular glue to bind COI1 and JAZ (Figure 1B). Detailed analyses of the interaction between the COI1-JAZ coreceptor and its ligands are essential for developing a COI1-JAZ agonist/antagonist that could potentially regulate plant defense.^15^^,^^16^^,^^17^ In 2018, a detailed mode of ligand perception by the COI1-JAZ co-receptor was demonstrated using isothermal titration calorimetry (iTC).^18^ Using iTC, a new model of ligand perception based on COI1-JAZ was proposed. Coronatine (COR; Figure 1A), a structural mimic of (3*R*, 7*S*)-JA-Ile,^12^^,^^19^ is perceived by COI1-JAZ through a unique two-step perception mode, in which COR is first perceived by COI1 and subsequently recruits JAZ to form a COI1-COR-JAZ ternary complex (Figure 1C).^18^ Since then, the two-step perception mode has been believed to be the universal mechanism of ligand perception by COI1-JAZ. However, because the iTC assay requires a large amount of COI1 protein, no other jasmonates, including the naturally occurring hormone (3*R*, 7*S*)-JA-Ile, were examined using iTC.^12^

In this study, we used the differential scanning fluorimetry (DSF) assay, also known as the thermal stability shift assay, to directly measure the affinity of ligands for the *Arabidopsis* COI1 protein because the DSF assay requires a lower amount of COI1 protein than the iTC assay.^20^ The DSF assay measures the shift in melting temperature, the temperature at which protein unfolding occurs, of the receptor protein with or without a ligand, and allows for direct observation of the affinity between the receptor protein and the non-labeled ligand.^21^ The DSF assay has been used for studying the interaction between strigolactone or abscisic acid and their receptors.^22^^,^^23^^,^^24^ The DSF assay has demonstrated that COR and COR-MO,^15^ which is potent antagonist of COI1-JAZ, are perceived by COI1-JAZ via a two-step perception mode. In contrast, in the current study, we demonstrate that the perception of the natural hormone (3*R*, 7*S*)-JA-Ile occurs through a one-step mode of COI1-(3*R*, 7*S*)-JA-Ile-JAZ complex formation and may not depend on the two-step mode. Thus, we conclude that the *Arabidopsis* COI1-JAZ co-receptor system has two independent modes of ligand perception.

## Results

### DSF assay can demonstrate the two-step ligand perception mode of COI1-JAZ co-receptor

**Figure 2:**
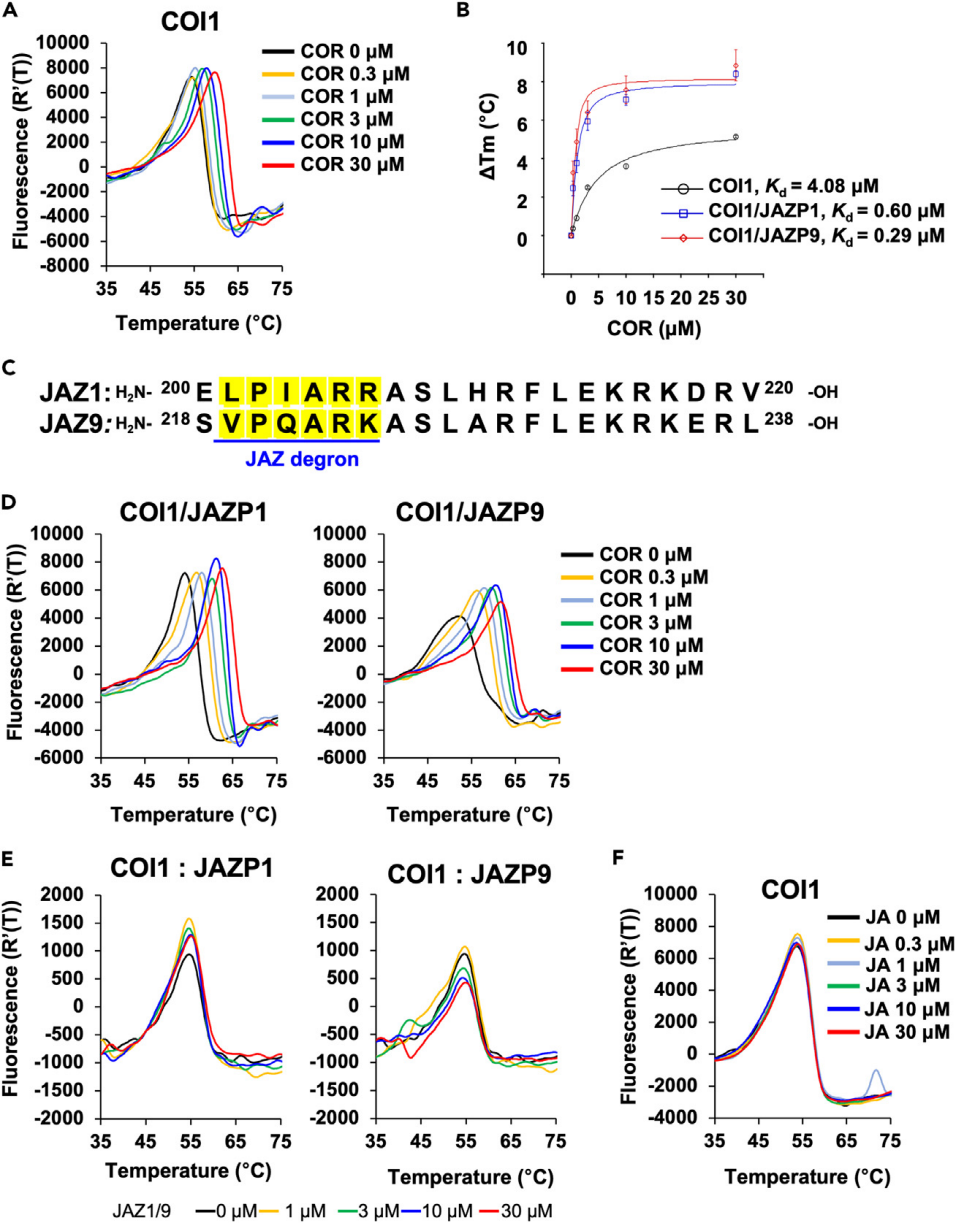
DSF assay of COR (A) Differential scanning fluorimetry (DSF) melting temperature curves of COI1 protein in the absence or presence of COR (0.3–30 μM). (B) ΔTm dose-response curves of COR. Experiments were performed in triplicate and data are shown as the mean ± S.D. (C) Chemical structures of JAZP1/9 degron peptides. JAZ degron sequences are highlighted in yellow. (D) DSF melting temperature curves of COI1 protein with JAZP1/9 in the absence or presence of COR (0.3–30 μM). (E) DSF melting temperature curves of COI1 protein in the absence or presence of JAZP1/9 (1–30 μM). (F) DSF melting temperature curves of COI1 protein in the absence or presence of JA (0.3–30 μM).

For the DSF assay, we prepared tag-free *Arabidopsis* COI1 protein from glutathione-*S*-transferase (GST)-fused COI1 co-expressed with *Arabidopsis* SKP1-like (ASK1) which improved the expression yield in Sf9 cultured insect cells.^25^ The GST tag was removed using the Tobacco Etch Virus (TEV) protease (Figure S1).^19^ First, we used COR as a positive control in the DSF assay because the direct interaction of COR with COI1 has been previously confirmed using the iTC assay.^18^ We examined the varying concentration of COI1 (0.1, 0.5, and 1.0 μM) and 1.0 μM condition showed a clear melting curve and was employed in the following assay (Figure S2A). The melting point of COI1 shifted to a higher temperature in the presence of COR (0.3–30 μM; Figures 2A and S3A), indicating the successful observation of COI1-COR interaction using DSF. The *K*_d_ value (Figure 2B; Table 1, *K*_d_ = 4.08 μM) observed through the DSF assay was four times larger than that previously reported using the iTC assay (*K*_d_ = 0.96 μM).^18^ As shown in Figures S2B and S2C, ASK1 did not affect the result of DSF assay because the background DSF curves of ASK1 were negligible compared to those of the COI1-ASK1 complex. The addition of JAZ1/9 degron peptide (JAZP1/9, Figures 2C and S4), which contains the short degron motif necessary for the interaction with the ligand, improved the *K*_d_ values (Figures 2B, 2D, S3B, and S3C) (Table 1, *K*_d_ = 0.60 μM for COI1-JAZ1 and *K*_d_ = 0.29 μM for COI1-JAZ9). On the other hand, no shift of melting point was observed by the addition of JAZP1/9 to COI1 (Figure 2E). This result suggests that the conformation of COI1, which is fixed by binding to the ligand, can be further tightened by adding JAZP1/9. The observed *K*_d_ values were consistent with the previously obtained *K*_d_ value for COI1-JAZP1 (*ca.* 0.11 μM).^19^ In contrast, no shift in the melting point of COI1 was observed upon addition of racemic jasmonic acid (Figure 1A), which was used as a negative control (Figures 2F and S5A).^10^^,^^18^ The addition of JAZP1/9 did not affect the shift in melting point (Figures S5B and S5C). We also examined (+)-*cis*-12-oxo-phytodienoic acid (*cis*-OPDA, Figure 1A) as a negative control,^10^ and no shift was observed in the melting point of COI1 upon titration (Figure S6). These results confirmed that the DSF assay provides reliable results on the interaction of COR and COI1/COI1-JAZs and demonstrated that the two-step perception mode can be proven by methods other than the iTC.

**Table 1:** The *K*_d_ values of ligands toward COI1/COI1-JAZP1/9 obtained from DSF assays

Ligand	*K*_d_ (μM)		
	COI1	COI1-JAZ1	COI1-JAZ9
COR	4.08	0.60	0.29
COR-MO	2.65	2.31	2.67
CFA-Ile	14.1	2.66	2.95
CFA-Val	28.1	6.63	9.77
(*3R*, *7S*)-JA-Ile	ND^a^	17.7	7.39
JA-CMA^b^	ND^a^	33.5	24.2

a not determined.

b (*3R*, *7R*): (*3R*, *7S*) = 95:5 mixture.

### COI1 perceives the COR-derived antagonist COR-MO via a two-step perception mode

**Figure 3:**
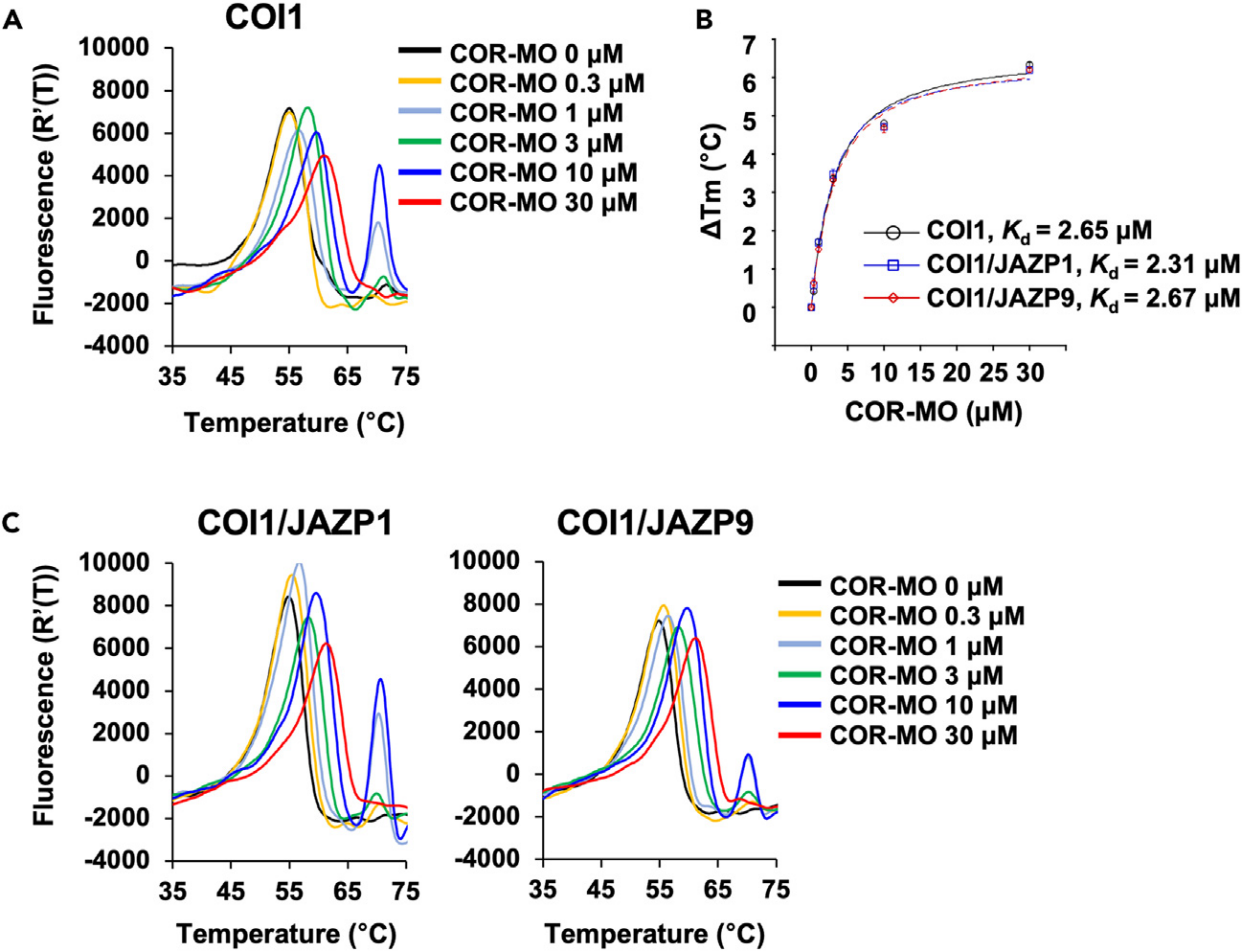
DSF assay of COR-MO (A) Differential scanning fluorimetry (DSF) melting temperature curves of COI1 protein in the absence or presence of COR-MO (0.3–30 μM). (B) ΔTm dose-response curves of COR-MO. Experiments were performed in triplicate and data are shown as the mean ± S.D. (C) DSF assays using COI1 and JAZP1/9 with COR-MO (0–30 μM).

COR-MO (Figure 1A), an *O*-methyl oxime derivative of COR, is a potent antagonist of the COI1-JAZ co-receptor.^15^ The putative mode of action of COR-MO is explained by a two-step perception mode: COR-MO potentially first binds to COI1 and then interferes with the recruitment of JAZ through steric hindrance caused by the *O*-methyl oxime group.^18^ This mode of action is strongly supported by molecular dynamics calculations and subsequent docking simulations.^26^ However, the proposed mode of action of COR-MO has not yet been confirmed experimentally. We examined the interaction between COR-MO and COI1/COI1-JAZ using the DSF assay. The melting point of COI1 shifted to a higher temperature in the presence of COR-MO (0.3–30 μM) (Figures 3A and S7A), and the *K*_d_ value for the COI1-COR-MO interaction was obtained (Figure 3B, *K*_d_ = 2.65 μM). *K*_d_ (COI1-COR-MO) was comparable to *K*_d_ (COI1-COR) (Table 1). In contrast to COR (Figure 2B), the addition of JAZP1/9 did not affect the *K*_d_ value (Figures 3B, 3C, S7B, and S7C), suggesting the inhibition of JAZ recruitment to the COI1-COR-MO complex. These results are consistent with the previously proposed two-step mode of ligand perception and demonstrate that COR-MO first binds to COI1 and inhibits JAZ access to the COI1-COR-MO complex.

### Affinity of the plant hormone (3*R*, 7*S*)-JA-Ile to COI1 and COI-JAZ1/9

**Figure 4:**
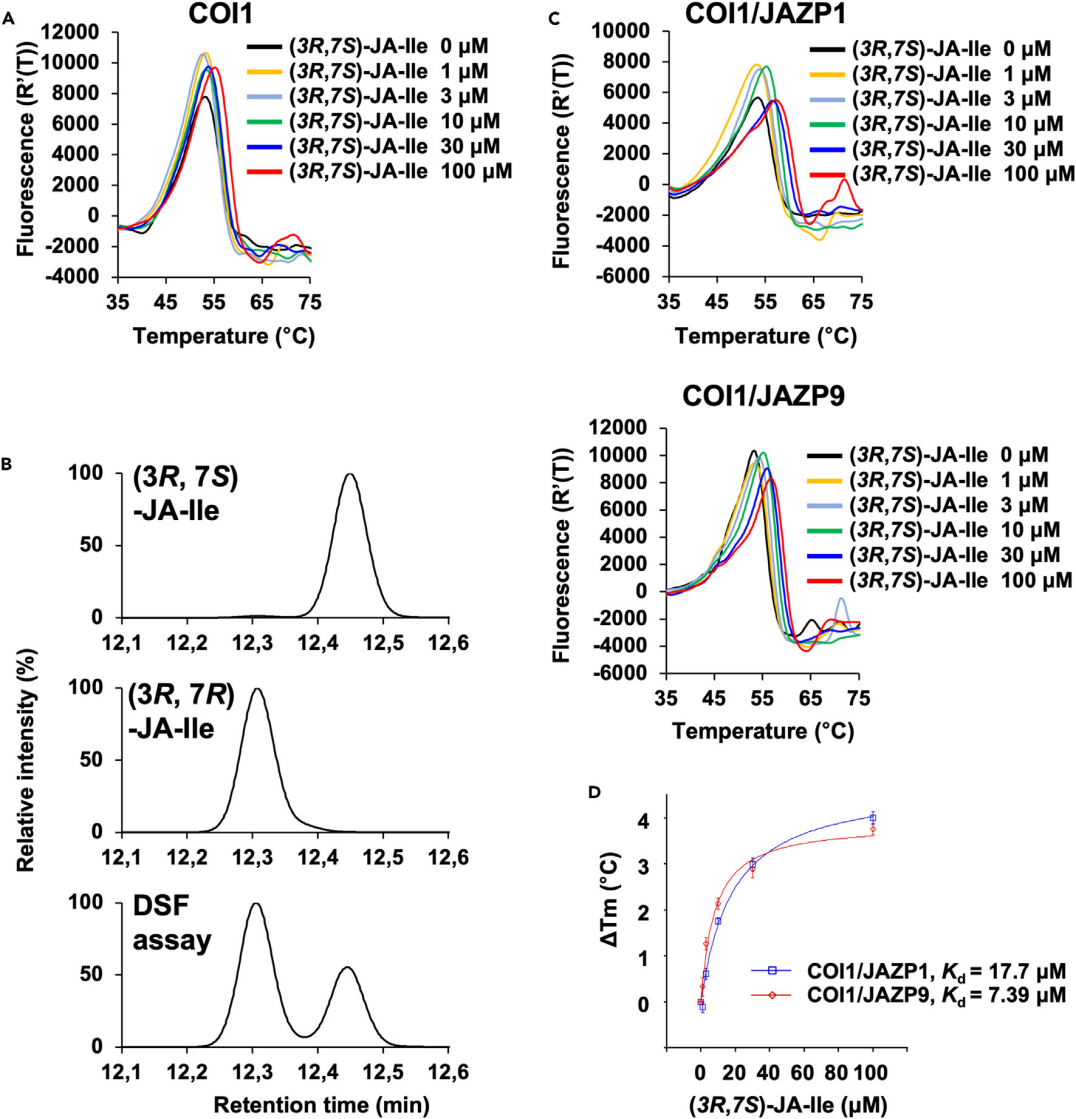
DSF assay of (3*R*, 7*S*)-JA-Ile (A) Differential scanning fluorimetry (DSF) melting temperature curves of COI1 protein in the absence or presence of (3*R*, 7*S*)-JA-Ile (1–100 μM). (B) The isomerization from (3*R*, 7*S*)-JA-Ile to (3*R*, 7*R*)-JA-Ile in the assay solution was analyzed by UPLC-MS/MS. (C) DSF melting temperature curves of COI1 protein with JAZP1/9 in the absence or presence of (3*R*, 7*S*)-JA-Ile (1–100 μM). (D) ΔTm dose-response curves of (3*R*, 7*S*)-JA-Ile. Experiments were performed in triplicate and data are shown as the mean ± S.D.

Next, we used the DSF assay to examine the interaction of the naturally occurring plant hormone (3*R*, 7*S*)-JA-Ile with *Arabidopsis* COI1/COI1-JAZs to determine whether the two-step perception mode applies to the endogenously working jasmonate system. (3*R*, 7*S*)-JA-Ile has a weaker affinity than COR for COI1-JAZ1/9.^12^ Therefore, we performed a DSF assay using a higher ligand concentration of (3*R*, 7*S*)-JA-Ile (100 μM). In the presence of a higher concentration of (3*R*, 7*S*)-JA-Ile, there was little or no shift in the melting point of COI1 (Figures 4A and S8A), suggesting no or negligible interactions between (3*R*, 7*S*)-JA-Ile and COI1. However, it should be noted that the active *cis*-isomer (3*R*, 7*S*)-JA-Ile may have isomerized to the inactive *trans*-isomer (3*R*, 7*R*)-JA-Ile during the DSF assay (Figure 1A).^12^ The *trans*-isomer (3*R*, 7*R*)-JA-Ile is thermodynamically more stable than the *cis*-isomer (3*R*, 7*S*)-JA-Ile, and heating of the sample solution during the DSF assay may have caused isomerization, leading to a significant decrease in affinity. To confirm this, the isomerization of (3*R*, 7*S*)-JA-Ile to (3*R*, 7*R*)-JA-Ile under DSF conditions was analyzed by UPLC-MS/MS (Figure 4B). Under these conditions, (3*R*, 7*S*)-JA-Ile was converted to a mixture of 36.1% (3*R*, 7*S*)-JA-Ile and 63.9% (3*R*, 7*R*)-JA-Ile (Figure 4B). Thus, some amount of (3*R*, 7*S*)-JA-Ile still exists under the DSF heating conditions. In addition, isomerized (3*R*, 7*R*)-JA-Ile has little affinity for COI1, even at high concentrations; a slight shift in the melting point of COI1 was observed upon the addition of (3*R*, 7*R*)-JA-Ile (Figure S9).

Meanwhile, the addition of JAZP1/9 significantly improved the *K*_d_ values (Figures 4C and 4 days, S8B and S8C, *K*_d_ = 17.7 μM for COI1-(3*R*, 7*S*)-JA-Ile-JAZP1, and *K*_d_ = 7.39 μM for COI1-(3*R*, 7*S*)-JA-Ile-JAZP9). The *K*_d_ (COI1-(3*R*, 7*S*)-JA-Ile-JAZP1/9) values were approximately 25–30 times higher than the *K*_d_ (COI1-COR-JAZP1/9) values (Figure 2, Figure 4B and 4D). This result is consistent with the previous results of the pull-down assay, in which COR had approximately 100 times stronger affinity for COI1-JAZ9 than (3*R*, 7*S*)-JA-Ile.^12^

**Figure 5:**
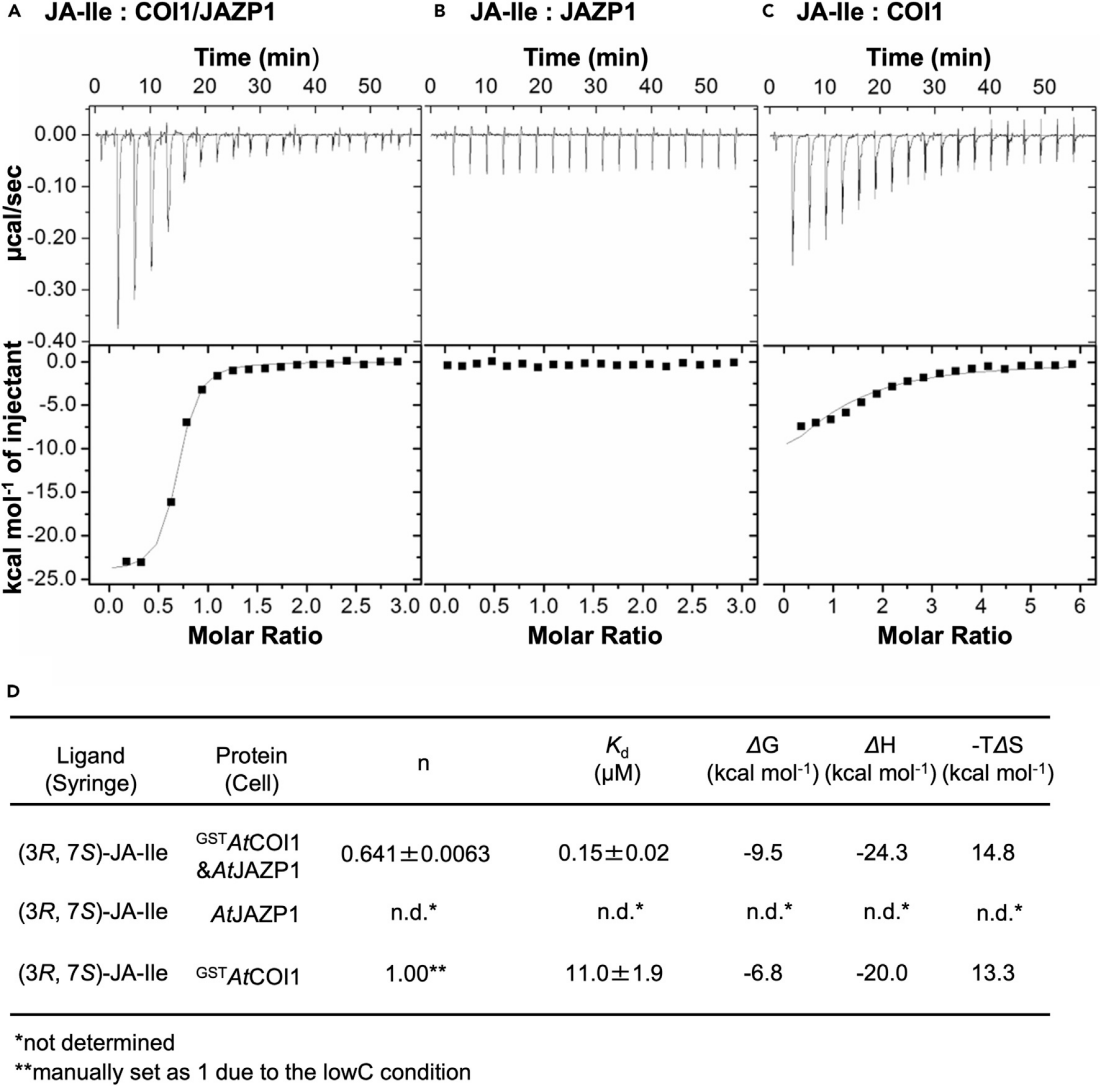
Thermodynamic analysis of the interaction between GST-COI1, (3*R*, 7*S*)-JA-Ile, and JAZP1 (A–C) iTC results of injecting (A) (3*R*, 7*S*)- JA-Ile (150 μM) into GST-COI1/JAZP1 (10 μM), (B) (3*R*, 7*S*)- JA-Ile (150 μM) into JAZP1 (10 μM), and (C) (3*R*, 7*S*)- JA-Ile (300 μM) into GST-COI1 (10 μM). (D) Thermodynamic parameters determined by ITC.

We also performed the iTC assay to validate the result of DSF assay. The iTC assay using GST-COI1, GST-COI1/JAZP1, JAZP1, and JA-Ile afforded distinct colorimetric titration curves, respectively (Figure 5). A complex of GST-COI1, JAZP1, and JA-Ile showed the clear sigmoidal curve to afford a strong *K*_d_ value of 0.15 μM (Figures 5A and 5D). JAZP1 and JA-Ile only showed the heat of dilution, suggesting no interaction between JAZP1-ligand (Figures 5B and 5D). GST-COI1 and JA-Ile showed a relatively gradual titration curve and required 6 eq. of JA-Ile for saturation to afford 11.0 μM of *K*_d_ value (Figures 5C and 5D), suggesting the existence of weak non-binding-pocket interaction and the interaction is negligible in the physiological conditions.^18^^,^^27^

### The structural factor determining the mode of ligand perception by COI1-JAZ

**Figure 6:**
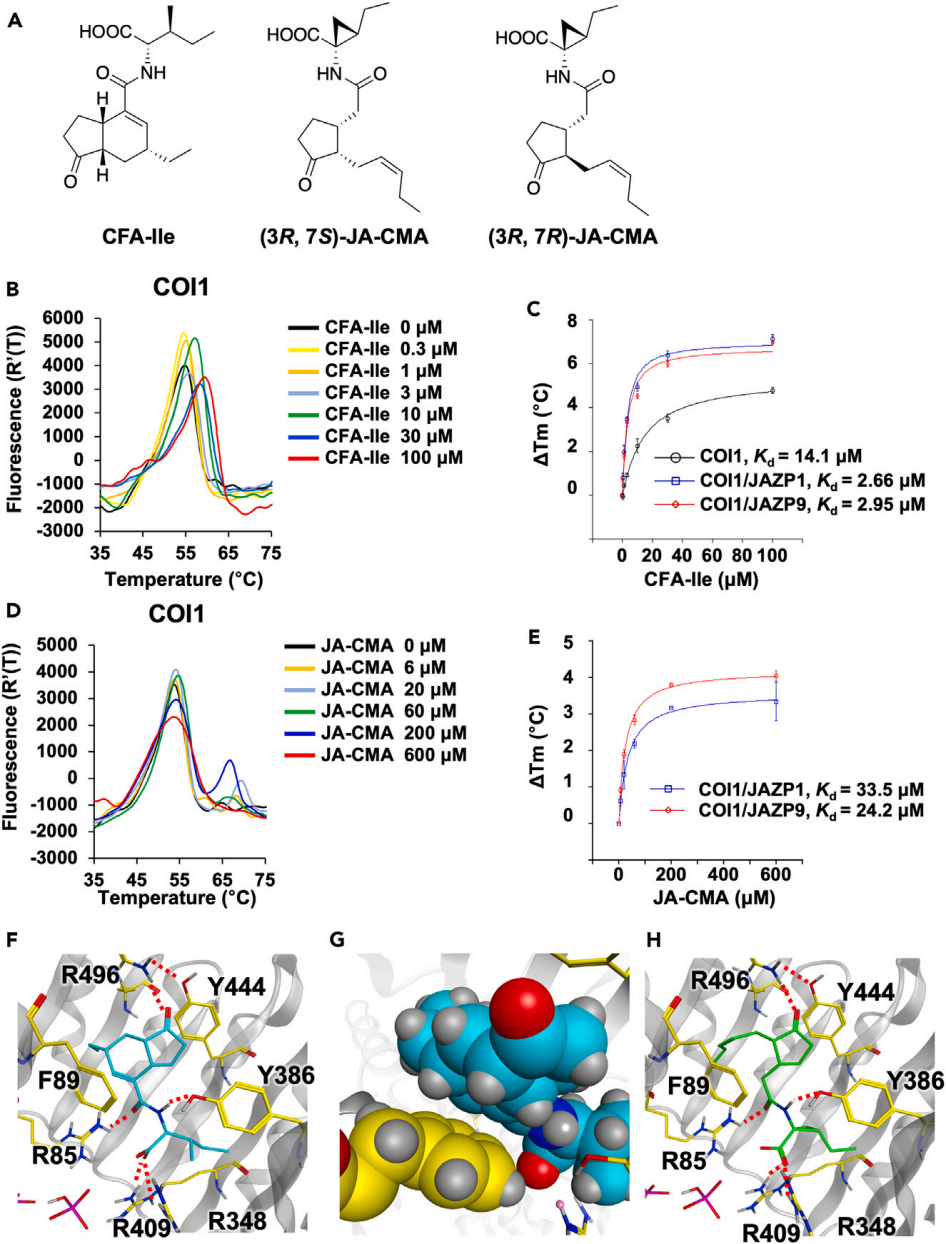
DSF assay and docking analysis of CFA-Ile and JA-CMA (A) Chemical structures of CFA-Ile and JA-CMA. (B) Differential scanning fluorimetry (DSF) melting temperature curves of COI1 protein in the absence or the presence of CFA-Ile (0.3–100 μM). (C) ΔTm dose-response curves of CFA-Ile. Experiments were performed in triplicate and data are shown as the mean ± S.D. (D) DSF melting temperature curves of COI1 protein in the absence or the presence of JA-CMA (6–600 μM). (3*R*, 7*R*)-JA-CMA: (3*R*, 7*S*)-JA-CMA = 95:5 mixture was used. (E) ΔTm dose-response curves of JA-CMA. Experiments were performed in triplicate and data are shown as the mean ± S.D. (F) Docking structure of the COI1-JAZ1 crystal structure (PDB: 3OGM) with CFA-Ile. Docking simulations of CFA-Ile (cyan) were conducted against the ligand binding site of COI1 (gray) and JAZ1 in the crystal structure (PDB: 3OGM). Red dash bonds indicate the potential hydrogen bond. (G) Expanded view of the interface of CFA-Ile (cyan) and Phe89 (yellow) in the docking structure of COI1/CFA-Ile/JAZ1. (H) Docking structure of the COI1-JAZ1 crystal structure (PDB: 3OGM) with (3*R*, 7*S*)-JA-CMA. Docking simulations of (3*R*, 7*S*)-JA-CMA (green) were conducted against the ligand binding site of COI1 (gray) and JAZ1 in the crystal structure (3OGM). Red dash bonds indicate the potential hydrogen bond.

To understand the structural factors contributing to the difference between the affinity of *Arabidopsis* COI1 to COR and (3*R*, 7*S*)-JA-Ile, the COI1 affinities of COR and (3*R*, 7*S*)-JA-Ile hybrid molecules were examined. COR is composed of (3*R*, 7*S*)-coronafacic acid (CFA) and (2*S*, 3*S*)-coronamic acid (CMA), with CFA structurally mimicking (3*R*, 7*S*)-jasmonic acid (JA) and CMA structurally mimicking L-Ile. Therefore, (3*R*, 7*S*)-coronafacyl L-Ile (CFA-Ile, Figure 6A)^28^^,^^29^ and (3*R*, 7*S*)/(3*R*, 7*R*)-jasmonoyl CMA (JA-CMA, Figure 6A) were synthesized as hybrid molecules (Figure S10). Their COI1 affinities were evaluated using DSF. We could not separate (3*R*, 7*S*)- and (3*R*, 7*R*)-JA-CMA by HPLC; thus, we used a 5/95 mixture of (3*R*, 7*S*)/(3*R*, 7*R*)-JA-CMA in the DSF assay (Figure S11). As expected, CFA-Ile caused a significant shift in the melting point in the DSF assay, and the *K*_d_ was calculated to be 14.1 μM (Figures 6B, 6C, and S12A; Table 1). Similar to the case of COR, the addition of JAZP1/9 improved the *K*_d_ values (Figures 6C, S12B and S12C) (Table 1, *K*_d_ = 2.66 μM for COI1-JAZP1 and *K*_d_ = 2.95 μM for COI1-JAZP9). In contrast, there was little or no shift in the melting point upon the addition of JA-CMA (Figures 6D and S13A; Table 1). Furthermore, adding JAZP1/9 improved the *K*_d_ values (Figures 6E, S13B, and S13C) (Table 1, *K*_d_ = 33.5 μM for COI1-JAZP1 and *K*_d_ = 24.2 μM for COI1-JAZP9). In addition, (3*R*, 7*S*)-coronafacyl L-Val (CFA-Val), which is known to bind with COI1-JAZ1 co-receptor,^28^^,^^29^ caused a significant shift in the melting point of COI1 and the addition of JAZP1/9 improved the *K*_d_ values as anticipated (28.1 μM for COI1, 6.63 μM for COI1-JAZP1 and *K*_d_ = 9.77 μM for COI1-JAZP9) (Table 1; Figure S14). These results demonstrated that the CFA moiety determines the ligand-binding mode, either one-step or two-step. To examine the role of the CFA moiety in binding, we performed docking simulations of CFA-Ile and JA-CMA with COI1. Sandwich-type interactions of the ligands with the two aromatic side chains of COI1, Phe89 and Tyr444, were observed (Figures 6F and 6G). There were no differences between the hydrogen-bonding networks of COI1-JAZ1, CFA-Ile, and JA-CMA (Figures 6F and 6H). It was estimated that the strength of the hydrophobic interactions would be much greater in CFA-Ile than in JA-CMA, considering that the cyclohexene ring of CFA was oriented face-to-face to the phenyl group of Phe89 (Figure 6G), which would contribute to the observed stronger affinity of CFA-Ile for COI1.

## Discussion

Various *in vitro* biochemical assays have been used to study the interactions between COI1-JAZ and its ligands.^20^^,^^30^ Most of these *in vitro* assays depend on the ligand-mediated formation of the COI1-ligand-JAZ ternary complex. These assays include pull-down,^10^^,^^11^ yeast-two-hybrid (Y2H),^10^ surface plasmon resonance (SPR),^18^^,^^31^ AlphaScreen,^32^ and fluorescence anisotropy.^33^ In contrast, the iTC assay, which measures the heat exchange during ligand-receptor complexation, does not depend on ternary complex formation and can monitor the direct interaction between the ligand and COI1 or JAZ. However, the iTC assay requires a large amount of COI1 protein; therefore, an assay method for COI1-ligand interactions that requires a low amount of COI1 protein is key.

In this study, we used the DSF assay, which requires a smaller amount of COI1 protein than iTC, to study the direct affinity of ligands for the *Arabidopsis* COI1 protein. DSF assays have been successfully used to analyze the receptor interactions of other plant hormones, such as abscisic acid and strigolactones.^22^^,^^24^^,^^34^ Using the DSF assay, we confirmed the two-step perception of COR by the COI1-JAZ coreceptor (Figures 2A, 2B, and 2D). To the best of our knowledge, this is the first successful application of DSF in the study of COI1-ligand interactions. Compared to previous results from iTC and FA assays,^18^^,^^33^ the DSF assay showed a larger *K*_d_ value for the COI1-JAZ co-receptor-ligand interaction, indicating a weaker evaluation of binding. Using the DSF assay, we also demonstrated the direct interaction of COR-MO, a potent antagonist of the COI1-JAZ co-receptor,^15^ and COI1, confirming the previously proposed but unconfirmed mode of action of this antagonist: COR-MO first binds COI1 and then perturbs the recruitment of JAZ, possibly because of steric hindrance due to the *O*-methyl oxime group (Figures 3A and 3B).^26^ In addition, the melting point of the COI1-COR-MO complex was not affected by the addition of JAZP (Figure 3C), indicating that the DSF assay can be used to efficiently screen for COI1-JAZ antagonists by comparing the melting points of COI1-ligand complexes with and without JAZP. The DSF assay is a powerful method for examining the direct affinity of a ligand for COI1 and provides valuable information for the development of a synthetic agonist/antagonist of the COI1-JAZ co-receptor system.

Our results suggest little or no affinity of (3*R*, 7*S*)-JA-Ile for COI1 (Figure 4A). Thus, unlike COI1-COR, COI1 alone may not function as a receptor for (3*R*, 7*S*)-JA-Ile in plant cells. The wound-induced accumulation of (3*R*, 7*S*)-JA-Ile in *Arabidopsis thaliana* is estimated to reach approximately 0.9–1.6 μM,^18^^,^^27^ and the local concentration of (3*R*, 7*S*)-JA-Ile in the nucleus, where COI1 is localized,^35^ is likely to be higher. However, these concentrations may not be sufficient to facilitate the interactions between COI1 and (3*R*, 7*S*)-JA-Ile. In addition, a previous study has reported that JA-Ile-MO (Figure 1A), the JA-Ile counterpart of COR-MO, does not function as a COI1-JAZ antagonist because of its extremely low inhibitory activity.^15^ These previously reported results are consistent with our conclusion that COI1 alone cannot perceive (3*R*, 7*S*)-JA-Ile to form a COI1–(3*R*, 7*S*)-JA-Ile complex for the subsequent recruitment of JAZ proteins. However, our current results also raise the possibility that the little or no affinity of (3*R*, 7*S*)-JA-Ile for COI1 could be due to the complete isomerization of the active *cis*-isomer (3*R*, 7*S*)-JA-Ile to the inactive *trans*-isomer (3*R*, 7*R*)-JA-Ile (Figure 1A), and we confirmed that (3*R*, 7*R*)-JA-Ile has little affinity for COI1 (Figure S9). UPLC-MS/MS analyses revealed that 36.1% of *cis*-isomer (3*R*, 7*S*)-JA-Ile remained stable under DSF conditions (Figure 4B). In a previous study, 65% of (3*R*, 7*S*)-JA-Ile was isomerized to (3*R*, 7*R*)-JA-Ile after 1 h of heating at 95°C^12^; this result is consistent with the results of the current study. However, the significant increase in *K*_d_ values when JAZP1/9 was added to the COI1-(3*R*, 7*S*)-JA-Ile complex suggests that complete isomerization and deactivation did not occur during the DSF assay. Thus, we evaluated the affinity of (3*R*, 7*S*)-JA-Ile for COI1 using the DSF assay. It was concluded that (3*R*, 7*S*)-JA-Ile is perceived by the COI1-JAZ co-receptor through a one-step formation of the ternary complex COI1-(3*R*, 7*S*)-JA-Ile-JAZ.

In conclusion, our results showed the *Arabidopsis* COI1-JAZ co-receptor system can perceive a ligand through a one- or two-step perception mechanism. Our results suggest that the two-step perception mode for interaction with COI1-JAZ and its ligands applies to COR and COR-based ligands. In contrast, the plant hormone (3*R*, 7*S*)-JA-Ile may be perceived by the COI1-JAZ co-receptor in a one-step mode. Thus, we demonstrate two distinct modes of action of molecular glue in the ligand perception of plant hormone co-receptors and provide scientific insight for developing novel molecular glues for regulating protein–protein interactions in living systems.

### Limitations of the study

In the present study, we focused on the COI1-JAZ co-receptor in *A. thaliana*. However, previous studies have suggested differences in ligand perception by COI1-JAZ homologs in other plant species such as *Solanum lycopersicum* and *Oryza sativa*.^32^^,^^36^^,^^37^ The modes of action of (3*R*, 7*S*)-JA -JA-Ile and COR in other plant species were not identified in the present study. Therefore, it remains unclear whether the conclusions of this study apply equally to COI1-JAZ co-receptors in other plant species. In particular, monocotyledonous plants, including *O. sativa,* have multiple *COI1* genes, and whether the modes of action of (3*R*, 7*S*)-JA-Ile and COR are the same requires further investigation. In addition, a unique co-evolution has occurred between the jasmonate ligands and the COI1-JAZ co-receptor. It would be interesting to determine whether the ligand perception mode of the receptor was preserved during evolution. In the primordial terrestrial plant *Marchantia polymorpha* and some mosses, ancestral jasmonates, dn-*cis*/*iso*-OPDA, function as molecular glues, forming a protein–protein interaction between *Mp*COI1 and *Mp*JAZ.^38^^,^^39^^,^^40^ The important question of whether the binding of dn-*cis*/*iso*-OPDA to the co-receptor *Mp*COI1-*Mp*JAZ follows a one-step or two-step mode was not addressed in this study and awaits further investigation. Detailed research from these perspectives will provide further insight into the mode of action of the COI1-JAZ ligand as a molecular glue.

## Methods

All methods can be found in STAR methods.

## STAR★Methods

### Key resources table

**Table:** 

REAGENT or RESOURCE	SOURCE	IDENTIFIER
**Chemicals, peptides, and recombinant proteins**		

List of ligands described in Figure 1, Figure 5A and 5A.	Refer to STAR Methods and Supplemental Information	N/A
D-myo-inositol-1,2,4,5,6-pentaphosphate, sodium salt	Cayman chemical	Cat #10008452
*At*JAZ1 degron peptide	Sheard et al., Nature 468, 400 (2010).^19^	N/A
*At*JAZ9 degron peptide	This paper	N/A
Glutathione Sepharose 4B	Cytiva	Cat#GE17-0756-01
Ni Sepharose^TM^ High Performance	Cytiva	Cat# 17-5268-02
TEV protease	New England BioLabs	Cat# P8112S
ESF921 medium	Funakoshi	Cat #96-001-01
PSFM-J1 Medium Wako, Liquid	wako	Cat #160-25851
FBS	biosera	Cat #556-33865
Recombinant *At*COI1-*At*ASK1	Sheard et al., Nature 468, 400 (2010).^19^ Takaoka et al., Nat. Commun. 9, 3654 (2018).^17^ Refer to STAR methods and Supplemental Information.	Addgene #29516 (pFB-GTE-COI1), #29517 (pFB-HTB-ASK1)

**Critical commercial assays**		

Protein Thermal Shift Dye Kit	Thermo Fischer Scientific	Cat#4461146 Lot: 01006761, 01288952
Micro BCA Protein Assay Kit	Thermo Fischer Scientific	Cat#23235 Lot: RB227004

**Experimental models: Cell lines**		

Sf9 insect cells	Oxford expression Technology	cat# 600100

**Software and algorithms**		

Protein Thermal Shift Software v1.4	Thermo Fischer Scientific	Cat#4466038
ChemDraw Professional 18.0	PerkinElmer	https://www.perkinelmer.com/category/chemdraw
MOE v2020.09	Chemical Computing Group	https://www.chemcomp.com/Products.htm
KaleidaGraph v4.1.1	Synergy Software	https://www.synergy.com/
Delta 6.1.0	JEOL	https://nmrsupport.jeol.com/
ChromNAV ver.2	JASCO	https://www.jasco.co.jp/jpn/product/ChromNAV/system-preparative.html
MicroCal iTC Origin	OriginLab	https://www.originlab.com/doc/ja/Quick-Help/View-OPJ-in-OldVer

**Other**		

StepOnePlus Real-Time PCR System	Thermo Fischer Scientific	https://www.thermofisher.com/jp/ja/home/life-science/pcr/real-time-pcr/real-time-pcr-instruments/step-one-real-time-pcr-systems.html
NanoPhotometer® N60	IMPLEN	https://www.implen.de/product-page/implen-nanophotometer-n60-microvolume-spectrophotometer
ÄKTA™explorer	Cytiva	https://www.cytivalifesciences.com/en/us/support/products/aktaexplorer-100-18111241
Amersham Imager 680 detector	Cytiva	https://www.cytivalifesciences.co.jp/catalog/43565.html
JNM-ECS-400 spectrometer	JEOL	https://www.jeol.co.jp/products/scientific/nmr/JNM-ECS.html
FT/IR-4100	JASCO	https://www.jasco.co.jp/jpn/product/FTIR/FTIR.html
microTOF II	Bruker Daltonics	N/A
Autoflex II	Bruker Daltonics	N/A
JASCO P-2200 polarimeter	JASCO	https://www.jasco.co.jp/jpn/product/Polarimeter/spec.html
Isolera^TM^ One	Biotage	https://www.biotage.co.jp/products_top/flash-purification/isolera_top/
Biotage® Initiator+ Alstra™	Biotage	https://www.biotage.com/alstra-peptide-synthesis
MicroCal iTC200	Malvern Panalytical	https://www.malvernpanalytical.com/jp/support/product-support/microcal-range/microcal-itc-range/microcal-itc200

### Resource availability

#### Lead contact

Minoru Ueda (minoru.ueda.d2@tohoku.ac.jp), Tohoku University, Sendai 980–8578, Japan.

#### Materials availability

Further information and requests for resources and reagents should be directed to and will be fulfilled by the lead contact, Minoru Ueda (minoru.ueda.d2@tohoku.ac.jp).

#### Data and code availability

(1) All data reported in this paper will be shared by the lead contact upon request.
(2) This paper does not report original code.
(3) Any additional information required to reanalyze the data reported in this paper is available from the lead contact upon request.

### Experimental model and subject details

#### Cell culture

Sf9 insect cells (Oxford Expression Technology) were grown as a suspension culture in ESF921 or PSFM-J1 medium supplemented with 2% fetal bovine serum (FBS), 0.5% penicillin-streptomycin, 0.5 ng/mL amphotericin B at 27°C, 127 rpm.

### Method details

#### General materials and methods

All chemical reagents and solvents were obtained from commercial suppliers (Wako Pure Chemical Industries Co. Ltd., Kanto Chemical Co., Inc., Tokyo Chemical Industry Co., Ltd., Nacalai Tesque Co., Ltd., Sigma-Aldrich Co. LLC., Watanabe Chemical Industries Co. Ltd., Thermo Fisher Scientific Inc., GE Healthcare) and used without further purification. Reversed-phase high–performance liquid chromatography (HPLC) was carried out on a PU–4180 plus pump equipped with UV–4075 and MD–4010 detectors (JASCO, Tokyo, Japan). Both ^1^H and ^13^C NMR spectra were recorded on a JNM–ECS–400 spectrometer (JEOL, Tokyo, Japan) in deuterated chloroform and using TMS as an internal standard. Fourier transforms infrared (FT/IR) spectra were recorded on an FT/IR–4100 (JASCO, Tokyo, Japan). High–resolution (HR) electrospray ionization (ESI)–mass spectrometry (MS) analyses were conducted using a microTOF II (Bruker Daltonics Inc., Billerica, MA). MALDI-TOF MS analysis was performed on an Autoflex II (Bruker Daltonics Inc., MA, US). Optical rotations were measured by a JASCO P–2200 polarimeter (JASCO, Tokyo, Japan). All anhydrous solvents were either dried by standard techniques and freshly distilled before use or purchased in anhydrous form. Flash chromatography was performed on the Isolera system (Biotage Ltd., North Carolina, US). TLC was performed on Silica gel F254 (0.25 mm or 0.5 mm, MERCK, Germany). All reactions were carried out under air unless stated otherwise. SDS-PAGE was performed using Mini-Protean III (Bio-Rad Laboratories, Inc., US) devices. CBB-stained gel images were obtained using the Amersham Imager 680 detector (GE Healthcare, CA, US). Protein concentration was determined using NanoPhotometerN60 (IMPLEN GmbH, Germany). DSF experiments were carried out using StepOnePlus Real-Time PCR System (Thermo Fischer Scientific Inc., MA US) equipped with Protein Thermal Shift Software (Thermo Fischer Scientific Inc., MA US). ITC experiments were carried out using iTC 200 Microcalorimeter (MicroCal, Malvern Panalytical, UK) equipped with Origin (version 7.0) software (OriginLab Co., MA, USA).

#### Chemical syntheses of COI1-JAZ ligands

We synthesized all of the chemical ligands of COI1-JAZ used in this study. Optically pure COR was synthesized according to our previous synthetic route.^41^^,^^42^ COR-MO was synthesized from COR according to the previously reported method.^15^ And *cis*-isomer (3*R*, 7*S*)-JA-Ile and *trans*-isomer (3*R*, 7*R*)-JA-Ile were obtained by the HPLC separation of synthetic mixture of (3*R*, 7*S*)-JA-Ile: (3*R*, 7*R*)-JA-Ile = 5 : 95 synthesized according to the previous reports.^12^ (3*R*, 7*S*)-coronafacyl L-Ile (CFA-Ile) was synthesized according to the previous report.^28^^,^^29^ and a novel compound jasmonoyl CMA (JA-CMA) was synthesized as follows: To a solution of JA (98.7 mg, 0.469 mmol) in THF (5.0 mL), Et_3_N (0.326 mL, 2.35 mmol) and ethyl chloroformate (0.051 mL, 0.47 mmol) were added. The mixture was stirred at room temperature for 1 h, and the resulting suspension was added to a solution of CMA⋅HCl (31.1 mg, 0.188 mmol) and diisopropylethylamine (0.065 mL, 0.37 mmol) in H_2_O (1.2 mL). The mixture was stirred at room temperature for 1 h and quenched with 1M aq. HCl. The resulting mixture was extracted with EtOAc. The combined organic layers were washed with brine, dried over Na_2_SO_4,_ and concentrated under reduced pressure. The crude product was purified by medium-pressure chromatography (Isolera, eluent: CHCl_3_/MeOH = 97/3 to CHCl_3_/MeOH = 70/30) to afford JA-CMA (22.9 mg, 38%) as a white solid. Synthesized JA-CMA was further purified by RP-HPLC (ODS-MG-5, 20 × 250 mm, Develosil) with 0.1% HCO_2_H solution (MeCN/H_2_O = 45/55) at 8.0 mL/min to give JA-CMA (Rt = 20 min). The obtained JA-CMA was estimated as (3*R*, 7*S*)-JA-CMA: (3*R*, 7*R*)-JA-CMA = 5 : 95 mixture from ^1^H-NMR.

^1^H-NMR (400MHz, CDCl_3_) δ_H_; 6.33 (brs, 1H), 5.45 (dt. *J* = 9.8, 7.1 Hz, 1H), 5.28 (dt. *J* = 9.8, 8.0 Hz, 1H), 2.89 (brs, 0.05H, *cis*-isomer), 2.59 (dd, *J* = 14.2, 3.7 Hz, 0.95H, *trans*-isomer), 2.41–2.20 (m, 5H), 2.06 (quintet. *J* = 7.6 Hz, 2H), 2.18–1.96 (m, 2H), 1.91 (dt. *J* = 10.1, 5.0 Hz, 1H), 1.67–1.49 (m, 4H), 1.44 (quintet, *J* = 7.3 Hz, 1H), 1.29–1.22 (m, 1H), 1.01 (t, *J* = 7.3 Hz, 3H), 0.96 (t, *J* = 7.6 Hz, 3H), COO*H* not observed; ^13^C-NMR (100MHz, CDCl_3_) δc: 219.3, 173.4, 134.1, 125.1, 54.0, 40.7, 38.6, 38.4, 37.7, 33.8, 27.0, 25.6, 22.7 20.6, 20.5, 14.1, 13.4, carboxyl *C*OOH not observed; IR (film) cm^−1^: 3308, 2965, 2933, 2875, 1734, 1698, 1654, 1540, 1457, 1276, 1179; HRMS (ESI, negative) m/z [M-H]^-^ calcd. for C_18_H_26_NO_4_: 320.1867, found: 320.1863; [α]_D_^23^ -4.72° (c 0.58, MeOH).

#### Preparation of tag-free COI1 protein

The full-length *Arabidopsis thaliana* COI1 and ASK1 were co-expressed as a GST fusion protein in Sf9 insect cells.^19^^,^^25^ GST-COI1 and ASK1 P2 virus were infected Sf9 insect cells (final 1 × 10^6^ cells/mL), which were cultured in 4 L of ESF921 medium matrix (2% FBS, 0.5% Penicillin-Streptomycin, 0.5 ng/mL Amphotericin B). After incubation for 72 h, the infected cells were enriched by centrifugation and subsequently lysed by sonication (Lysis buffer: 20 mM Tris-HCl, 200 mM NaCl, 10% Glycerol, 1 mM dithiothreitol, 1xcOmplete Protease inhibitor EDTA free, pH 8.0). Insoluble components and microsome fractions were removed by centrifugation (1000 g, 4°C, 30 min) and ultracentrifugation (150000 g, 4°C, 1.5 h), respectively. 3.2 mL Glutathione Sepharose 4B (Cytiva, 80% resins in 4.0 mL EtOH, which was prewashed twice by 16 mL lysis buffer) was used to purify GST-COI1 protein in the cell lysate supernatant. The mixture was rotated for 1.5 h at 4°C and washed four times using a lysis buffer. The protein was solubilized in elution buffer (50 mM Tris-HCl, 100 mM NaCl, 10% Glycerol, 1 mM dithiothreitol, 0.1% Tween 20, 10 mM glutathione, pH 8.0). The first fraction was eluted by rotation for 20 min. This was followed by five times additional elution, and the supernatant was recovered by centrifugation. The obtained GST-COI1 fractions were concentrated into 2–3 mL by extra filtration using Amicon Ultra 15 (NMWL 10 kDa, Merck Millipore) (5000 g at 4°C). In iTC assay, this GST-COI1 solution was used with additional dialysis. For DSF assay, the solution of GST-COI1 was then transferred to Spectra/Por®Dialysis Membrane (Spectrum Laboratories, Inc.) and was treated with TEV protease (New England BioLabs) in the 1 L dialysis buffer (20 mM Tris-HCl, 200 mM NaCl, 10% Glycerol, 1 mM dithiothreitol, pH 8.0) for ca. 12 h at 4°C to cleave the GST tag and to remove glutathione. The equilibrated resin and protein solutions were rotated for 1.5 h at 4°C to remove the GST components, leaving tag-free COI1 protein in the supernatant. The supernatant was recovered by centrifugation (1000 g, 4°C, 1 min). The resin was washed once, then the supernatant was recovered by centrifugation.

The obtained crude fraction of tag-free COI1 was concentrated into <1 mL by extra filtration using Amicon Ultra 4 (NMWL 10kDa) (7500 g, 4°C). The concentrated fraction was purified by gel filtration chromatography on the AKTA system (Amersham Pharmacia Biotech, column: Hiload 16/60 Superdex 200 prep grade (1CV = 120 mL), elution buffer: 10 mM HEPES, 150 mM NaCl, pH 7.4, flow rate: 0.50 mL/min, detection: 280, 254, and 220 nm, fraction volume: 2.0 mL, equilibration: 1.1 CV, elution 1.2 CV). The fractions of pure COI1-ASK1 were collected, and the concentration was determined by UV absorption at 280 nm using NanoPhotometerN60 (IMPLEN GmbH, Germany).

#### Preparation of tag-free ASK1 protein

The full-length *Arabidopsis thaliana* ASK1 was expressed as a His-tag-fusion protein (His-ASK1) in Sf9 insect cells. ASK1 P2 virus was infected Sf9 insect cells (final 1 × 10^6^ cells/mL, MOI 1.5), which were cultured in 500 mL of PSFM-J1 medium matrix (2% FBS, 0.5% Penicillin-Streptomycin, 0.5 ng/mL Amphotericin B). After incubation for 72 h, the infected cells were enriched by centrifugation and subsequently lysed by sonication (Lysis buffer: 20 mM Tris-HCl, 200 mM NaCl, 10% Glycerol, 1 mM dithiothreitol, 1xcOmplete Protease inhibitor EDTA free, pH 8.0). Insoluble components and microsome fractions were removed by centrifugation (1000 g, 4°C, 30 min) and ultracentrifugation (150000 g, 4°C, 1.5 h), respectively. 0.5 mL Ni Sepharose High Performance (Cytiva, prewashed twice by lysis buffer) was used to bind His-ASK1 protein in the cell lysate supernatant. The mixture was rotated for 1.5 h at 4°C and washed seven times using a lysis buffer. The tag-free ASK1 protein was solubilized by treating the resin with TEV protease (New England BioLabs) in the lysis buffer (20 mM Tris-HCl, 200 mM NaCl, 10% Glycerol, 1 mM dithiothreitol, pH 8.0) for ca. 22 h at 4°C to cleave the His tag.

The obtained crude tag-free ASK1 was further purified by gel filtration chromatography on the AKTA system (Amersham Pharmacia Biotech, column: Hiload 16/60 Superdex 200 prep grade (1CV = 120 mL), elution buffer: 10 mM HEPES, 150 mM NaCl, pH 7.4, flow rate: 0.50 mL/min, detection: 280, 254, and 220 nm, fraction volume: 2.0 mL, equilibration: 1.1 CV, elution 1.2 CV). The fractions of pure ASK1 were collected, and the concentration was determined by UV absorption at 280 nm using a calculated extinction coefficient value of 0.783.

#### Syntheses and purification of JAZ peptide

JAZ1/9 were prepared using the fully automated microwave peptide synthesizer Initiator + Alstra (Biotage Ltd, North Carolina, US) starting from Fmoc-Val/Leu-Wang resin. The resin was swollen in DMF at 70°C for 20 min. The Fmoc protecting group was removed by treating with 20% piperidine in DMF twice. Amino acid coupling was accomplished by mixing the resin with Fmoc-protected amino acids (6 eq), 1-(7-aza-1H-benzotriazol-1-yl)-*N, N, N′, N′*-tetramethyluronium hexafluorophosphate (HATU, 6 eq), 1-Hydroxy-7-azabenzotriazole (HOAt, 6 eq), and DIPEA (12 eq) in DMF, and subjecting it to microwave irradiation at 50°C for either 30 min (Fmoc-Arg(Pbf)-OH) or 10 min (all others). After the peptide had been fully elongated, the resin was treated with 20% piperidine in DMF for 10-15 min to remove Fmoc. Then the peptide was deprotected with a cleavage cocktail (95% TFA, 2.5% TIS, 2.5% H_2_O) at r.t. for 2 h. The crude products were purified by HPLC using a Develosil ODS-HG-5 column (Φ 4.6 × 250 mm) eluting with a linear gradient (CH_3_CN (0.05% TFA): H_2_O (0.05% TFA) = 15:85 (5 min) to 50:50 (40 min) for JAZ1/CH_3_CN (0.05% TFA): H_2_O (0.05% TFA) = 20:80 (5 min) to 50:50 (35 min) for JAZ9) to afford JAZ9 peptide. After lyophilization, the peptide was dissolved in sterilized water to prepare the stock solution. The purity of each peptide was confirmed by HPLC analyses, and these were characterized by MALDI-TOF MS as follows; JAZ1: *m/z* [M + H]^+^ calcd for 2590.52, found 2590.51; JAZ9: *m/z* [M + H]^+^ calcd for 2483.47, found 2483.46.

#### Differential scanning fluorometry (DSF) assay

COR/COR-MO/CFA-Ile were dissolved in DMSO, (*3R*,*7S*)/(*3R*,*7R*)-JA-Ile and *cis*-OPDA were dissolved in ethanol, JA was dissolved in sterilized water to generate 10 mM stock solutions and diluted with corresponding solvent for preparation of 100 μM and 1 mM stock solutions, respectively. JAZ1/9 peptides were dissolved in sterilized water to generate 10 mM stock solutions and diluted to prepare 100 μM stock solutions. The concentration of tag-free COI1 was determined by UV absorption at 280 nm using a calculated extinction coefficient value of 0.962. DSF experiments were carried out using StepOnePlus Real-Time PCR System (Thermo Fisher Scientific, Inc., MA, US). Protein Thermal Shift Dye (Thermo Fisher Scientific, Inc., MA, US) was used as the reporter dye. Reaction mixtures were prepared in PCR tubes, and each reaction was carried out on a 20 μL scale in HEPES buffer (50 mM HEPES-NaOH buffer, 100 mM NaCl, 20 mM 2-mercaptoethanol, 10% glycerol, 5 μM inositol-1,2,4,5,6-pentakisphosphate (IP5), pH 7.8) containing 1 μM tag-free COI1 protein, 3 μM JAZ peptide and designated concentration of ligand. Samples were heated from 25 °C to 95 °C. The denaturation curve was obtained using Protein Thermal Shift Software (Thermo Fisher Scientific, Inc., MA, US) to afford ΔTm value for each condition. An average ΔTm value of three independent measurements was plotted, and ΔTm dose-response curves were analyzed with nonlinear curve-fitting analysis to evaluate *K*_*a*_ and *K*_*d*_ values using KaleidaGraph software (v4.1.1, Synergy Software).

#### Isothermal titration calorimetry (iTC) assay

The experiments were performed with iTC 200 Microcalorimeter (MicroCal, Malvern Panalytical, UK) at 25°C. GST-COI1 concentration was determined by BCA assay (Thermo Fisher Scientific, Inc., MA, US) using NanoPhotometerN60 (IMPLEN GmbH, Germany). (3*R*, 7*S*)-JA-Ile (150 μM or 300 μM from 10 mM DMSO stock solution) in the reaction buffer (50 mM Tris, 100 mM NaCl, 10% Glycerol, 0.1% Tween 20, 10 μM IP5, pH 7.8) was loaded into the syringe and titrated against a mixture of JAZP1 only (10 μM from 1 mM stock solution) or GST-COI1 only (10 μM) or GST-COI1/JAZP1 (10 μM) in the reaction buffer in the cell. The measurements were carried out by 19 × 2 μL successive injections of solutions (initial injection of 0.4 μL), with a 180s spacing time between injections, stirring of 750 rpm, and a reference power of 5 μcal/s. The measured heat changes of the binding reactions were integrated and processed using the standard “one set of sites” model implemented in the Origin (version 7.0) software package to determine the binding stoichiometry (n) and the equilibrium dissociation constant (*K*_d_) and other thermodynamic parameters (ΔG, ΔH, ΔS). Measurement between JA-Ile and GST-COI1 was analyzed with N = 1 manually due to the low C condition (C = n・M_tot_・*K*_a_, n: stoichiometry, M_tot_: molar concentration of sample molecules in the cell, *K*_a_: association constant).

#### In-*silico* docking analysis

The structural preparation program MOE 2020.09 (Chemical Computing Group) was used to deduce the structures of the absent residues in the crystal structure of the *At*COI1-JA-Ile-*At*JAZ1 complex (PDB ID: 3OGL). The docking structure of *At*COI-CFA-Ile/JA-CMA-*At*JAZ1 was prepared by replacing JA-Ile with CFA-Ile/JA-CMA in the docking simulation. A docking program in MOE was used for docking and Amber10:ETH force field parameters were assigned for the score estimations.

### Quantification and statistical analysis

#### Curve fitting analysis of ΔTm dose-response curve

The determination of the *K*_d_ value was conducted by curve fitting the dose-response ΔTm values. Experiments were performed in triplicate to obtain the mean and S.D. (shown as error bars). Dose-response curves were analyzed with the nonlinear curve-fitting analysis to evaluate apparent *K*_a_ and *K*_d_ values using KaleidaGraph v4.1.1 software.
