# Supplementary material for: Two distinct modes of action of molecular glues in the plant hormone co-receptor COI1-JAZ system
Source: iScience. 2023 Dec 3;27(1):108625. doi: 10.1016/j.isci.2023.108625 (PMC10770490; doi:10.1016/j.isci.2023.108625)

iScience, Volume *27*

Supplemental information

Two distinct modes of action of molecular glues in the plant hormone co-receptor COI1-JAZ system

Takuya Kaji, Kotaro Matsumoto, Taichi Okumura, Misuzu Nakayama, Shunji Hoshino, Yousuke Takaoka, Jianxin Wang, and Minoru Ueda

Supporting Information

Title:

Two distinct modes of action of molecular glues in the plant hormone co-receptor COI1-JAZ of *Arabidopsis thaliana*

Authors:

Takuya Kaji,[1] Kotaro Matsumoto,[1] Taichi Okumura,[1] Misuzu Nakayama,[1] Shunji Hoshino,[2] Yousuke Takaoka,[1] Jianxin Wang,[1] and Minoru Ueda[1,2]*

Affiliations:

[1] Department of Chemistry, Graduate School of Science, Tohoku University, Sendai 980-8578, Japan

[2]Department of Molecular and Chemical Life Sciences, Graduate School of Life Science, Tohoku University, Sendai 980-8578, Japan

Corresponding author and lead contact:

Minoru Ueda (minoru.ueda.d2@tohoku.ac.jp)

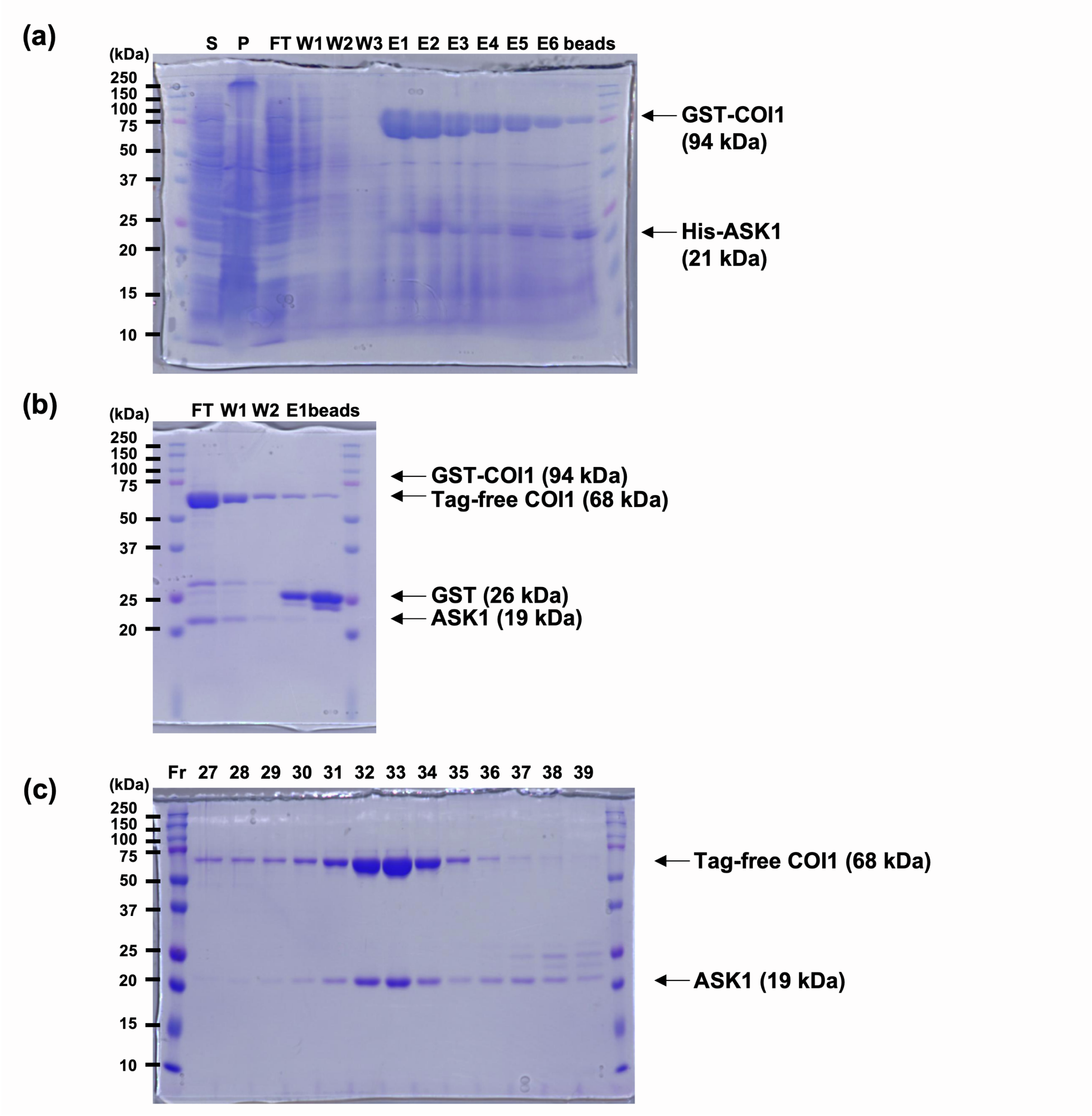

Figure S1. Expression and purification of GST-COI1 protein by cultured insect cell protein, related to Figures 2, 3, 4, 5, and 6. (a) SDS-PAGE analysis of GST-COI1 protein expressed by cultured insect cell protein expression system (S: soluble fraction of cell lysate, P: insoluble fraction (pellet) of cell lysate, FT: flowthrough fraction, W: washed fraction, E: elution fraction in glutathione-S-transferase affinity column chromatography, respectively). (b) Removal of GST tag using TEV-protease and purification of tag-free COI1. (c) Tag-free COI1 was purified by gel filtration chromatography, and fractions of pure tag-free COI1 were collected.

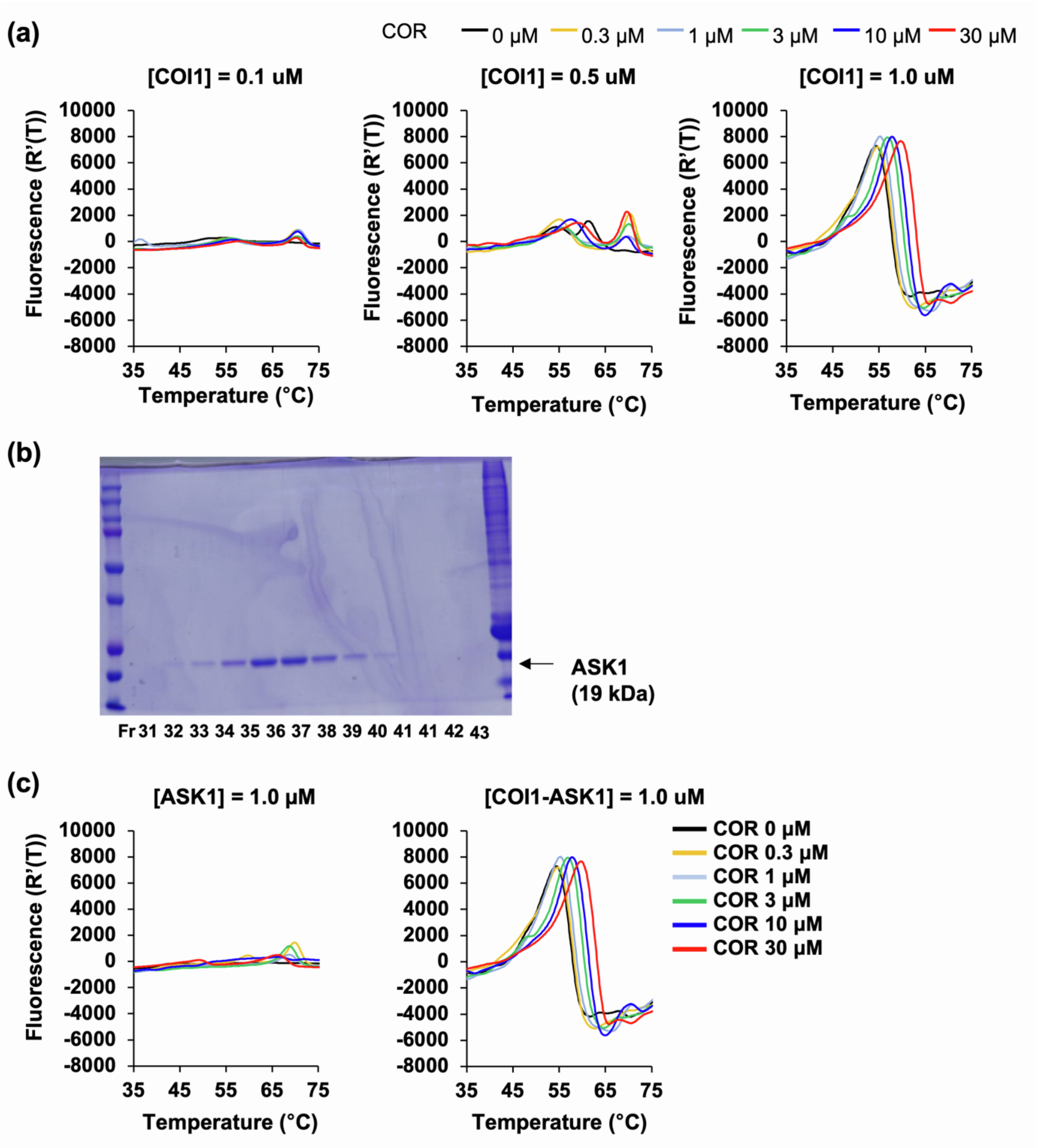

Figure S2. The DSF analyses of COI1-ASK1 protein, related to Figures 2. (a) The DSF melting temperature curves of COI1-ASK1 protein (COI1) in the different concentration (0.1, 0.5, 1.0 μM). (b) Expression and purification of ASK1 protein by cultured insect cell protein expression system (c) The DSF melting temperature curves of 1.0 μM ASK1 and 1.0 μM COI1-ASK1 in the absence or the presence of COR (0.3 to 30 μM).

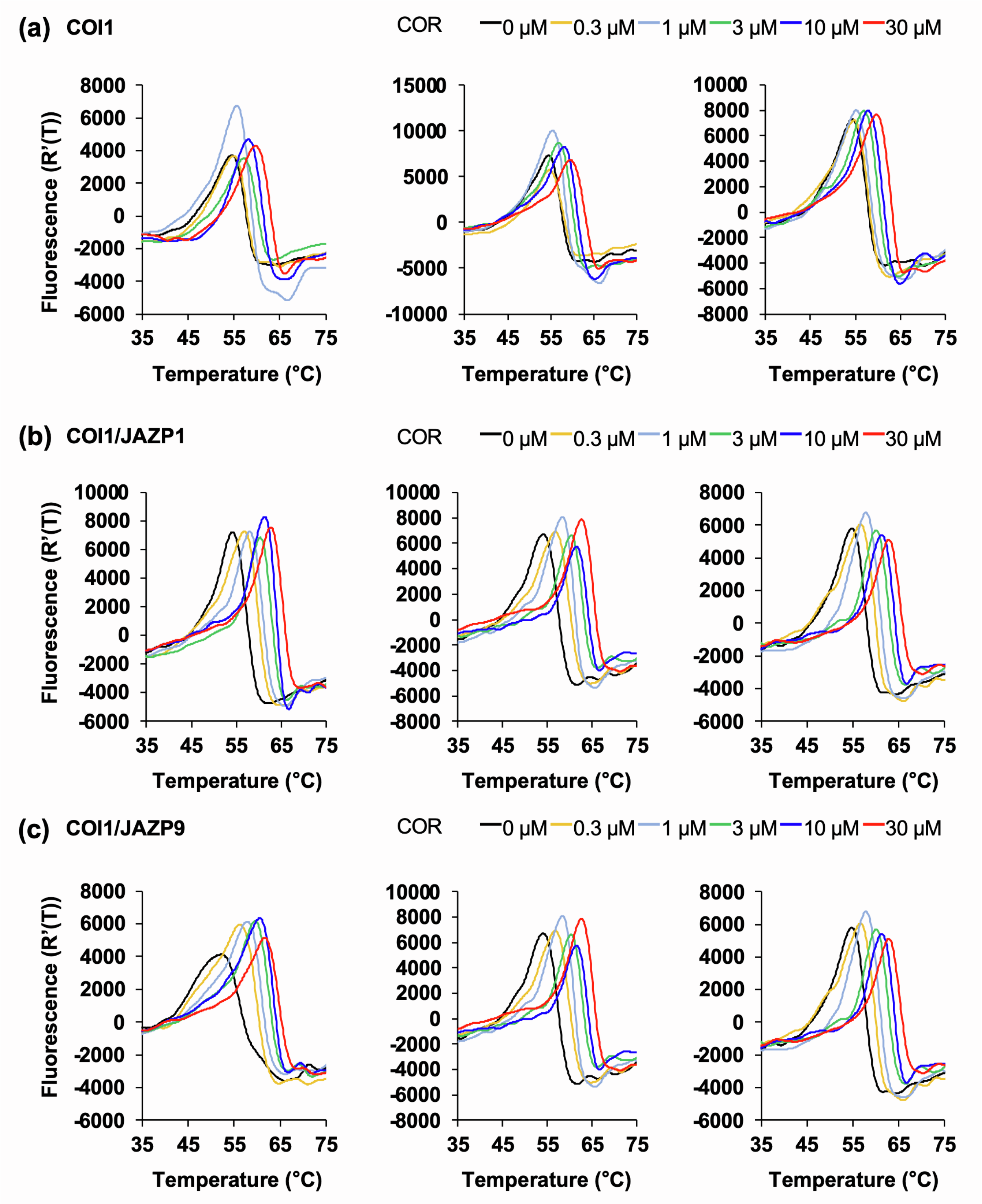

Figure S3. Triplicate of the DSF melting temperature curves of COI1 protein in the absence or the presence of COR (0.3 to 30 μM), related to Figures 2. (a) COI1, (b) COI1-JAZP1, (c) COI1-JAZP9.

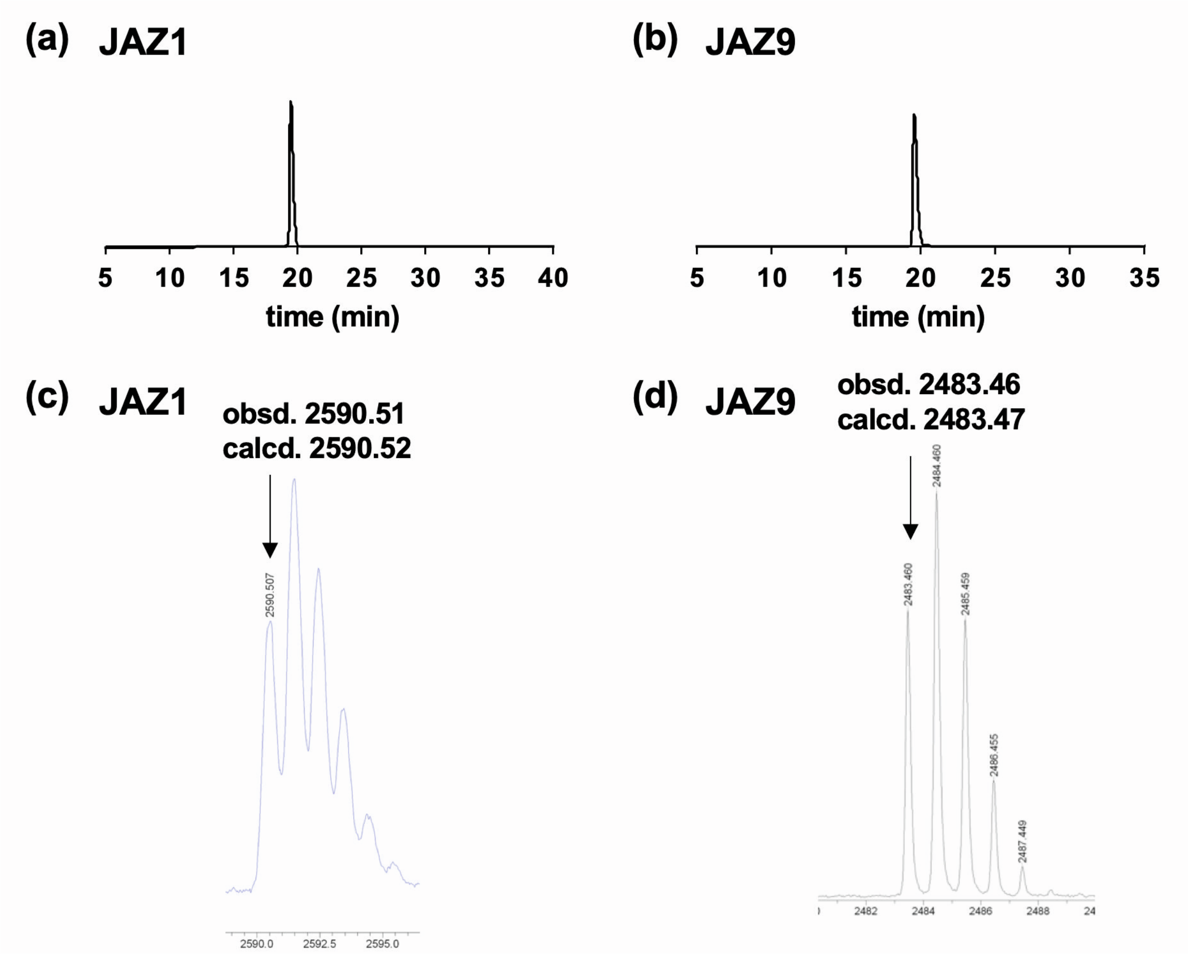

Figure S4. HPLC charts of purified JAZ peptides, related to Figures 2, 3, 4, 5, and 6. (a) JAZ1, (b) JAZ9. MALDI-TOF mass spectra of JAZ peptides; (c) JAZ1, (d) JAZ9.

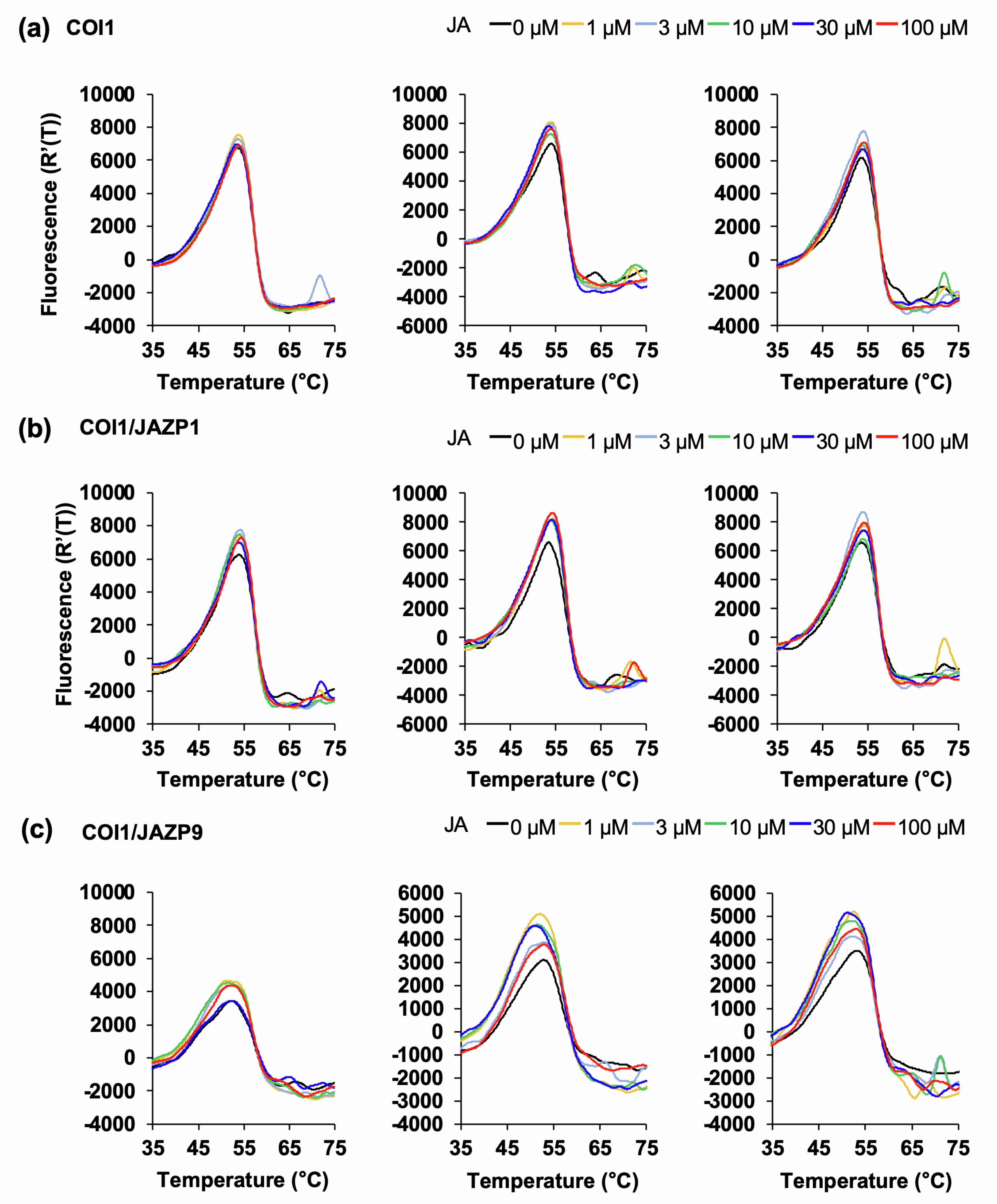

Figure S5. Triplicate of the DSF melting temperature curves of COI1 protein in the absence or the presence of JA (1 to 100 μM), related to Figures 2. (a) COI1, (b) COI1-JAZP1, (c) COI1-JAZP9.

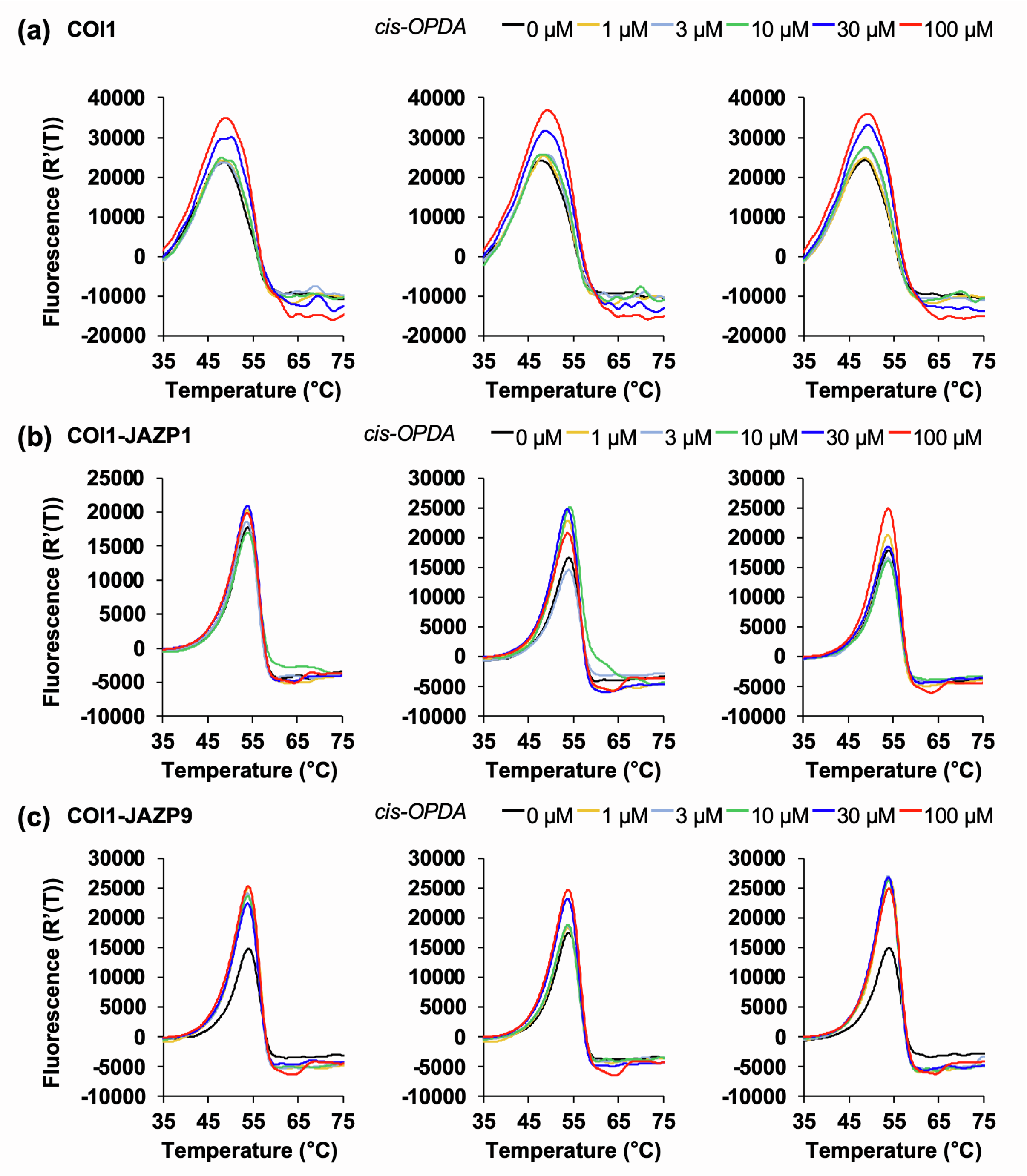

Figure S6. Triplicate of the DSF melting temperature curves of COI1 protein in the absence or the presence of *cis*-OPDA (1 to 100 µM) , related to Figures 2. (a) COI1, (b) COI1-JAZP1, (c) COI1-JAZP9.

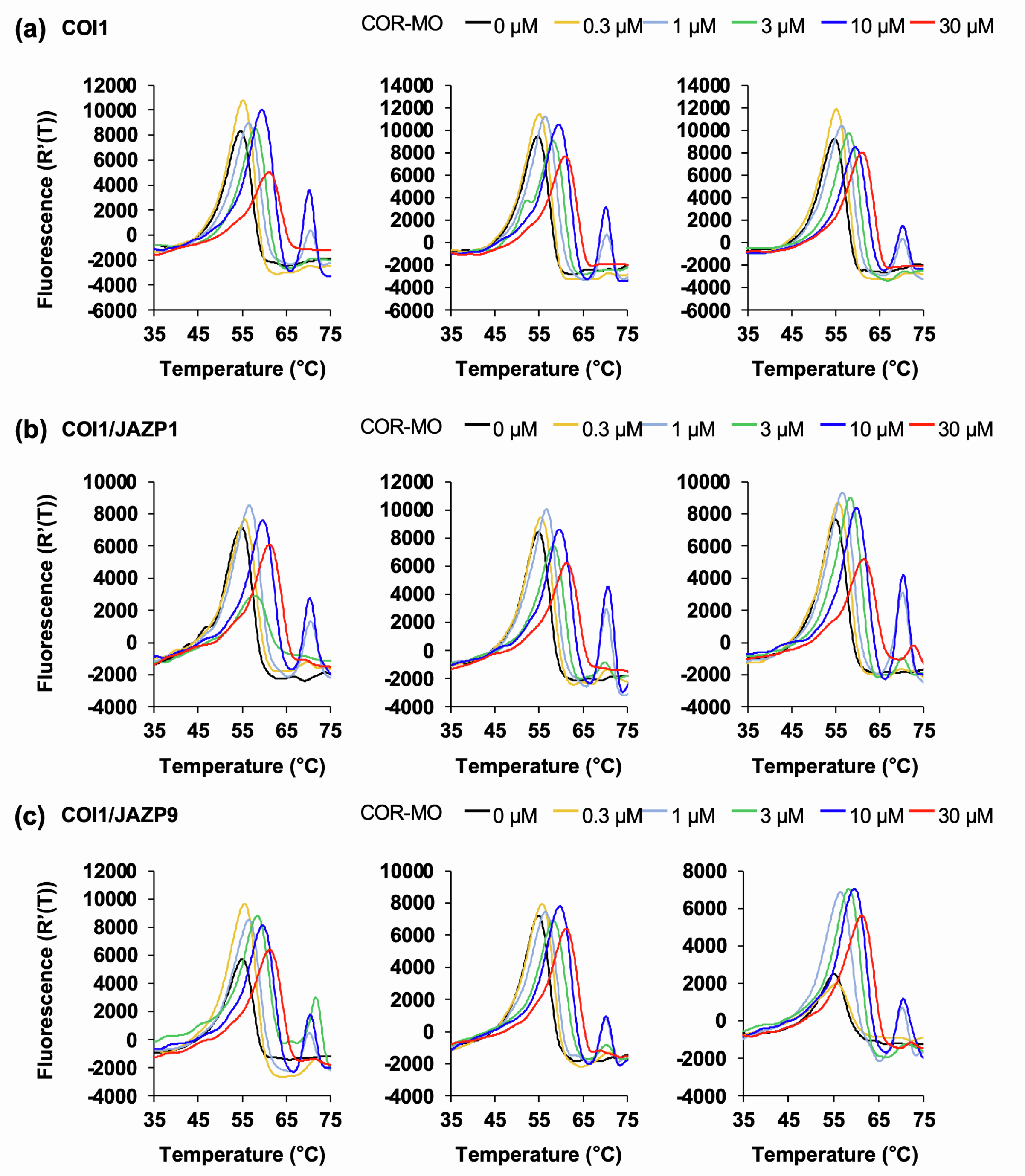

Figure S7. Triplicate of the DSF melting temperature curves of COI1 protein in the absence or the presence of COR-MO (0.3 to 30 µM), related to Figures 3. (a) COI1, (b) COI1-JAZP1, (c) COI1-JAZP9.

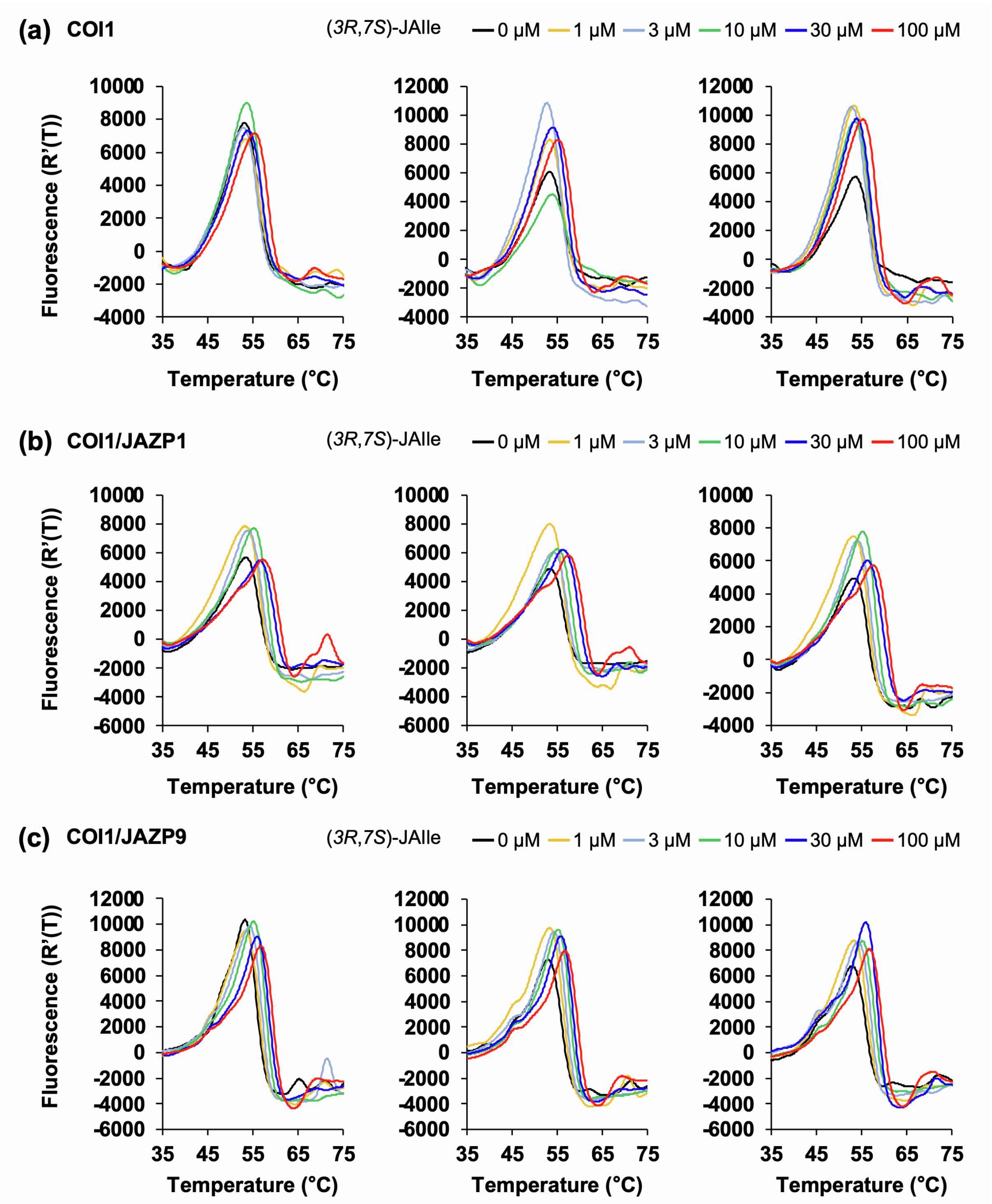

Figure S8. Triplicate of the DSF melting temperature curves of COI1 protein in the absence or the presence of (3*R*,7*S*)-JA-Ile (1 to 100 μM), related to Figures 4. (a) COI1, (b) COI1-JAZP1, (c) COI1-JAZP9.

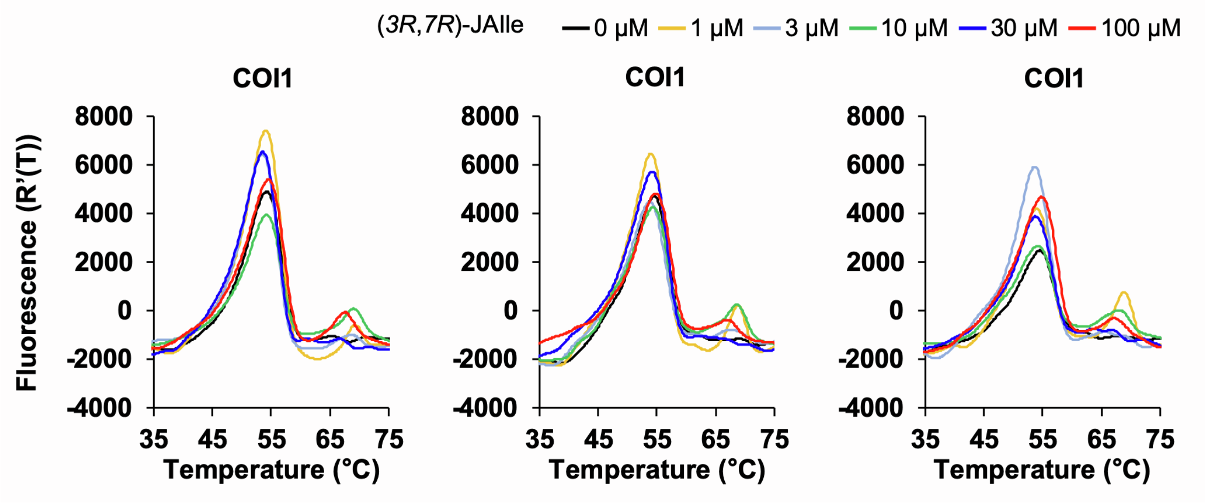

Figure S9. Triplicate of the DSF melting temperature curves of COI1 protein in the absence or the presence of (3*R*,7*R*)-JA-Ile (1 to 100 μM), related to Figures 4.

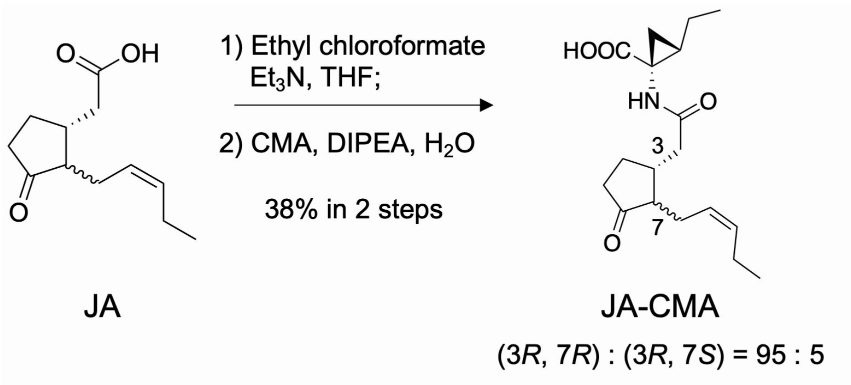

Figure S10. Chemical synthesis of JA-CMA, related to Figures 6.

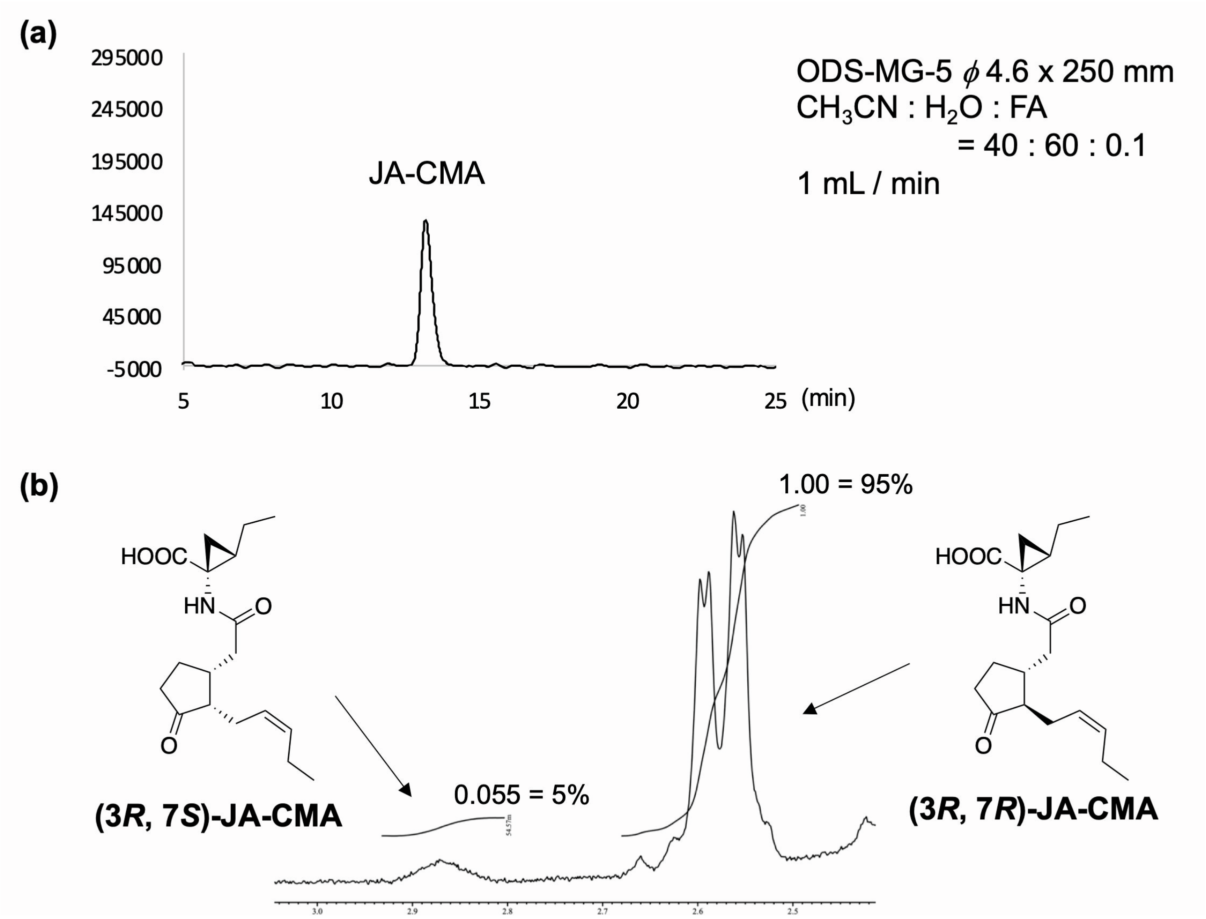

Figure S11. RP-HPLC analyses of synthetic JA-CMA, related to Figures 6. (a) Analytical RP-HPLC chromatogram of obtained JA-CMA, (b) Expanded view of ^{1}H-NMR showing that the synthesized JA-CMA was (3*R*, 7*R*) : (3*R*, 7*S*) = 95: 5 mixture.

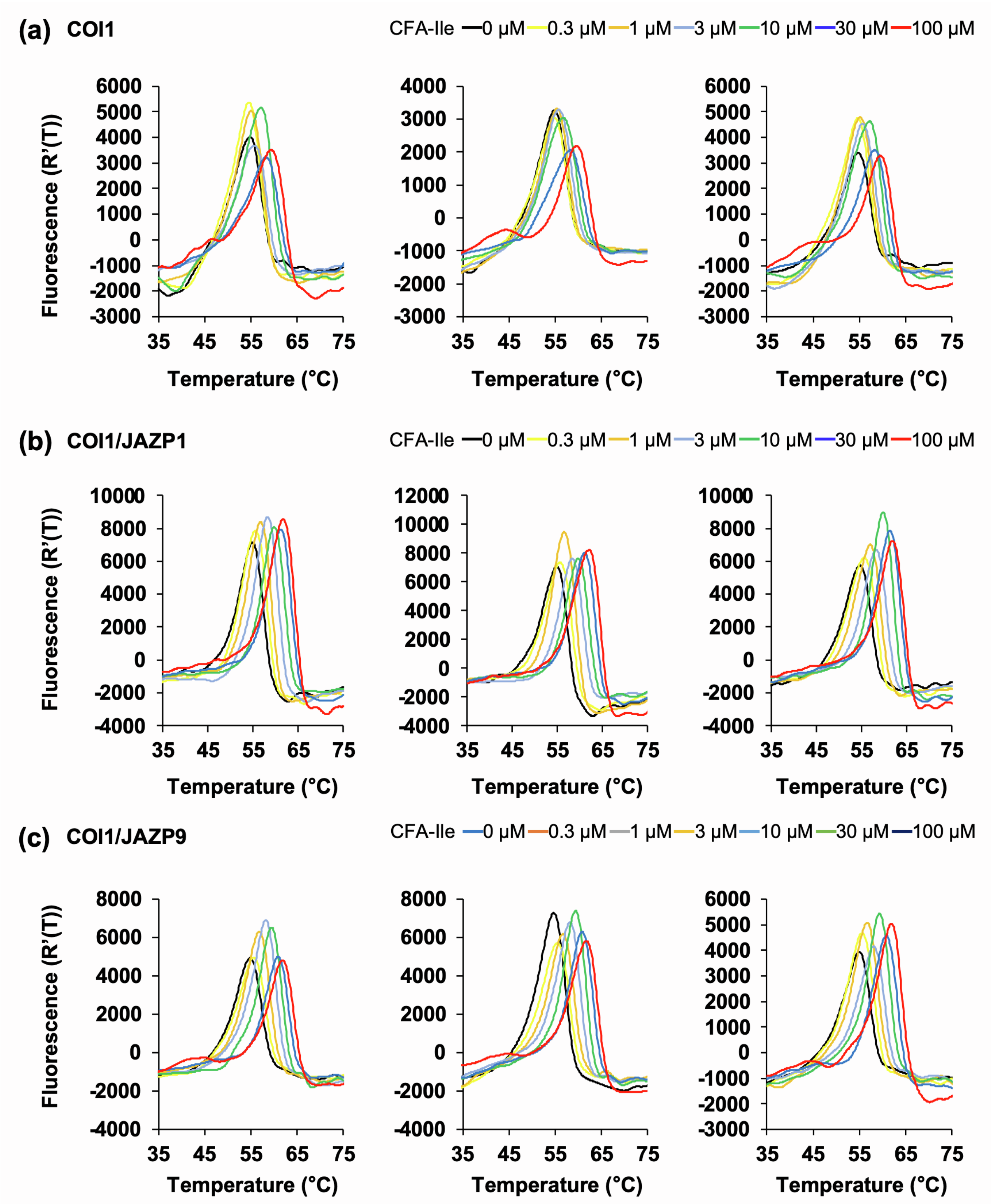

Figure S12. Triplicate of the DSF melting temperature curves of COI1 protein in the absence or the presence of CFA-Ile (0.3 to 100 μM), related to Figures 6. (a) COI1, (b) COI1-JAZP1, (c) COI1-JAZP9.

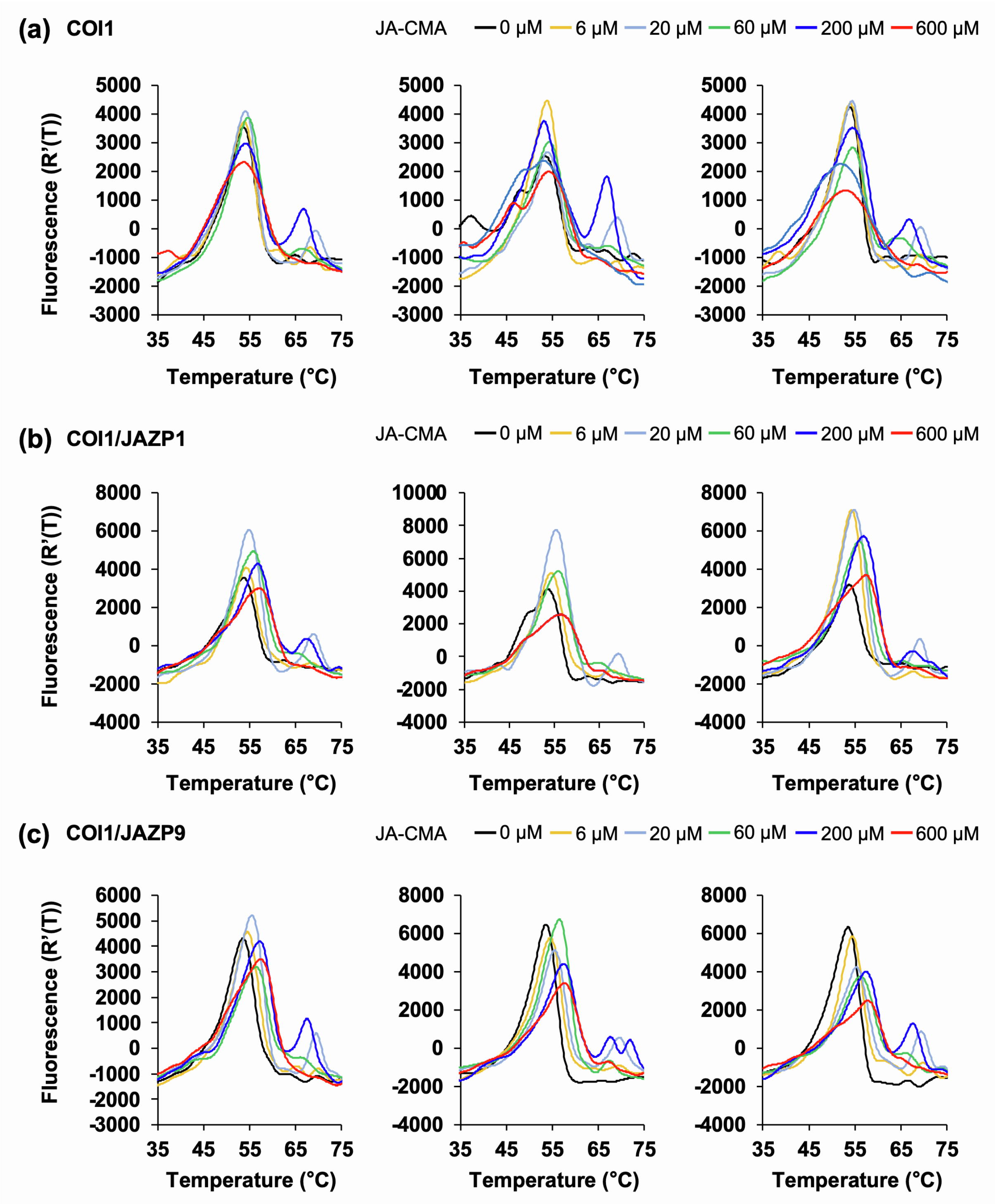

Figure S13. Triplicate of the DSF melting temperature curves of COI1 protein in the absence or the presence of JA-CMA (6 to 600 μM), related to Figures 6. (a) COI1, (b) COI1-JAZP1, (c) COI1-JAZP9.

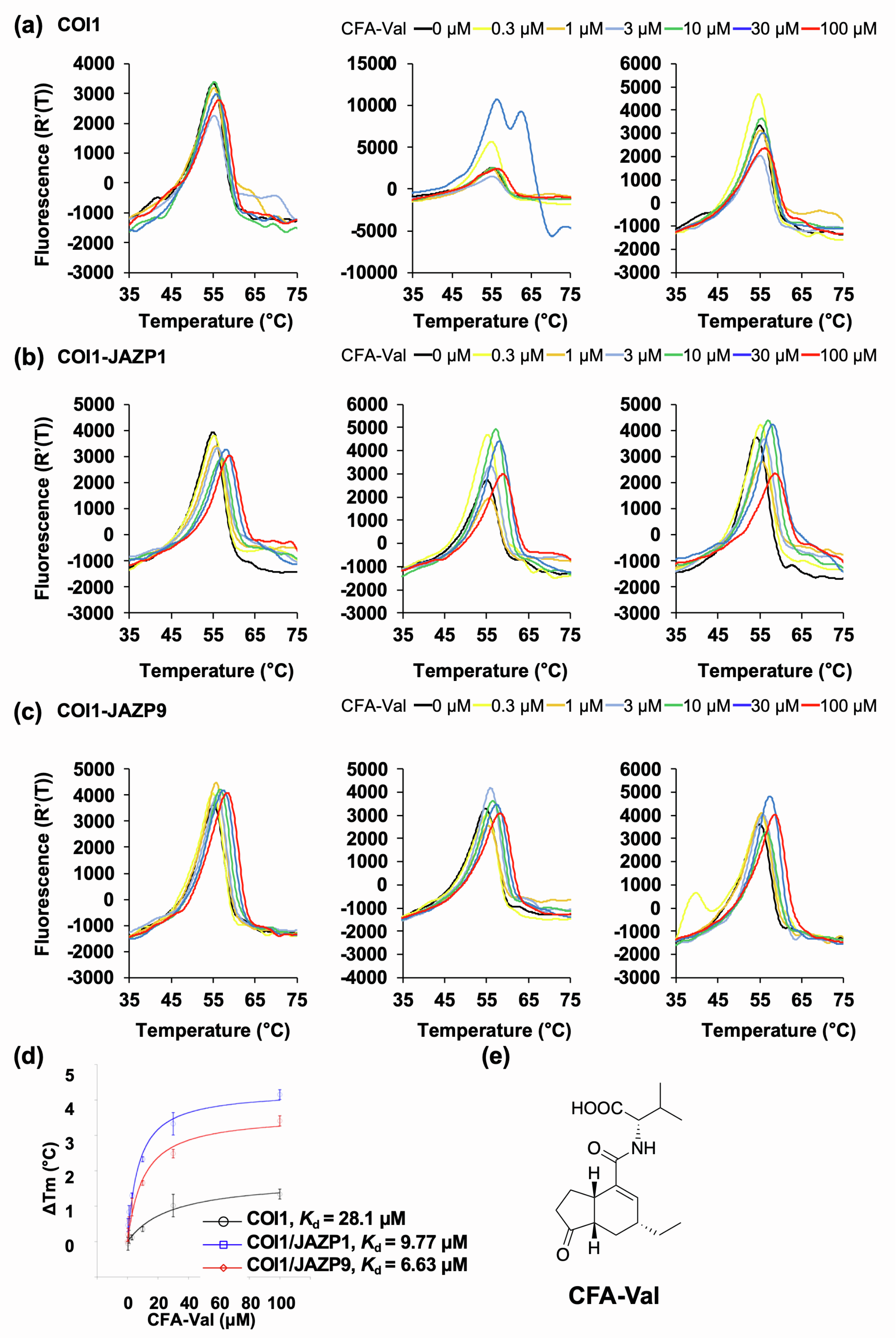
(a) COI1
CFA-Val —0 μM —0.3 μM —1 μM —3 μM —10 μM —30 μM —100 μM
Fluorescence (R'(T))
Temperature (°C)
(b) COI1-JAZP1
CFA-Val —0 μM —0.3 μM —1 μM —3 μM —10 μM —30 μM —100 μM
Fluorescence (R'(T))
Temperature (°C)
(c) COI1-JAZP9
CFA-Val —0 μM —0.3 μM —1 μM —3 μM —10 μM —30 μM —100 μM
Fluorescence (R'(T))
Temperature (°C)
(d)
ΔTm (°C)
CFA-Val (μM)
COI1, Kd = 28.1 μM
COI1/JAZP1, Kd = 9.77 μM
COI1/JAZP9, Kd = 6.63 μM
(e)
HOOC
NH
O
H
H
O
CFA-Val

Figure S14. The DSF analyses of interactions between COI1/COI1-JAZ and CFA-Val (0.3 to 100 μM), related to Figures 6. Triplicate of (a) COI1, (b) COI1-JAZP1, (c) COI1-JAZP9. (d) ΔTm dose-response curves of CFA-Val. Experiments were performed in triplicate to obtain mean and S.D. (shown as error bars). (e) Structure of CFA-Val.

Data S1. Spectra of a new compound JA-CMA, related to Figures 6, S10, and S11.

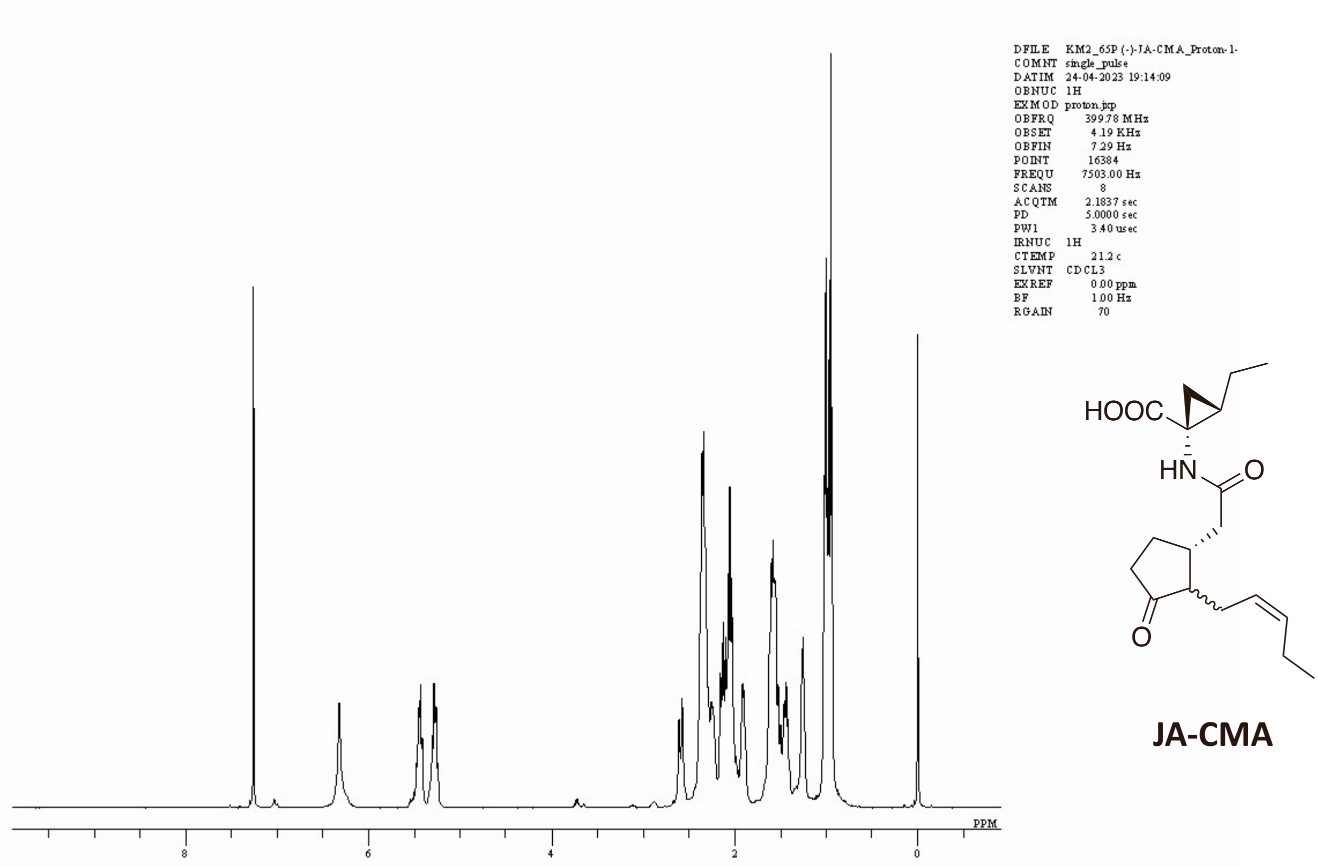

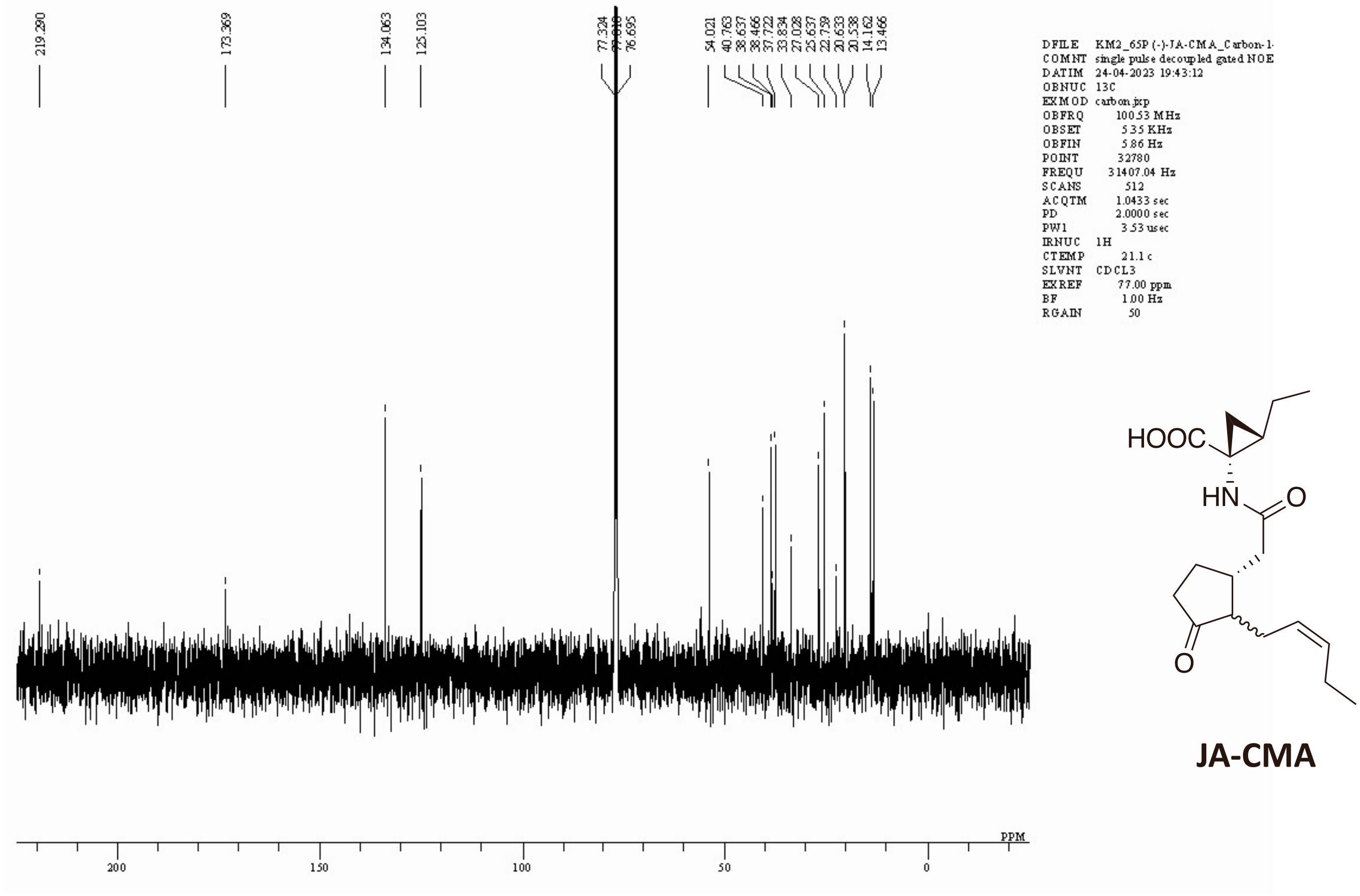

Supplement: Document S1. Figures S1–S14 and Data S1 [file mmc1.pdf]
